# The effect of circulating iron on barrier integrity of primary human endothelial cells

**DOI:** 10.1038/s41598-023-44122-6

**Published:** 2023-10-06

**Authors:** M. C. Madsen, F. Podieh, M. C. Overboom, A. Thijs, M. den Heijer, P. L. Hordijk

**Affiliations:** 1 Department of Physiology, Amsterdam UMC, De Boelelaan 1118, Amsterdam, 1081 HV Netherlands; 2 Department of Internal Medicine, Amsterdam UMC, Amsterdam, Netherlands; 3 Center of Expertise on Gender Dysphoria, Amsterdam UMC, Amsterdam, Netherlands

**Keywords:** Cell biology, Endocrinology, Cardiovascular biology, Circulation

## Abstract

Iron is hypothesized to be one of the contributors to cardiovascular disease and its levels in the circulation may correlate with cardiovascular risk. The aim of this study is to investigate the mechanisms that underlie the effects of iron on the barrier function of primary human endothelium. We used Human Umbilical Vein Endothelial Cells (HUVEC) to investigate the effects of Fe^3+^ using electric cell-substrate impedance sensing, microscopy, western blot and immunofluorescence microscopy. Exposure to Fe^3+^ caused EC elongation and upregulation of stress-induced proteins. Analysis of barrier function showed a dose-dependent drop in endothelial integrity, which was accompanied by Reactive Oxygen Species (ROS) production and could partly be prevented by ROS scavengers. Inhibition of contractility by the ROCK inhibitor Y27632, showed even more effective rescue of barrier integrity. Using western blot, we detected an increase in expression of the small GTPase RhoB, an inducer of EC contraction, and a small decrease in VE-cadherin, suggestive for an iron-induced stress response. Co-stimulation by TNFα and iron, used to investigate the role of low-grade inflammation, revealed an additive, negative effect on barrier integrity, concomitant with an upregulation of pro-inflammatory markers ICAM-1 and RhoB. Iron induces a response in HUVEC that leads to endothelial activation and a pro-inflammatory state measured by loss of barrier integrity which can be reversed by ROS scavengers, combined with inhibition of contractility. These data suggest that ROS-mediated damage of the vascular endothelium could contribute to the increased cardiovascular risk which is associated with elevated levels of circulating iron.

## Introduction

The role of iron in the body is two-faced; on the one hand it is essential for a range of physiological functions such as oxygen transport, mitochondrial activity, electron transport and DNA synthesis^1,2^. On the other hand, iron overload is toxic due to the generation of free radicals^3^. Iron is present in different forms: it can be incorporated into red blood cells, stored as ferritin, or it can circulate in the blood plasma. The majority of circulating iron is bound to transferrin, a carrier protein, but a small fraction of iron circulates in its free form as non-transferrin bound iron (NTBI). It is known that this NTBI can induce cell and tissue damage as it induces formation of reactive oxygen species (ROS) through the Fenton reaction^3^.

In 1981, Sullivan formulated his iron hypothesis in which he proposed a role of iron in the sex-associated differences in cardiovascular risk. In this hypothesis, women are protected against cardiovascular risk until menopause due to lower iron levels, which is caused by menstrual loss of iron. In contrast, men show higher circulating levels of iron and hemoglobin due to testosterone^4^. Since then, extensive research has been performed, accompanied by an ongoing debate regarding this topic, with some studies supporting the iron hypothesis while others did not find a role for iron in cardiovascular risk^5^. In general, laboratory and in vitro studies support the iron hypothesis and in vivo studies report contradictory results^6–8^.

The vascular endothelium is the inner lining of all blood vessels. The endothelium is directly exposed to all agents that are present in the circulation and is therefore also exposed to different forms of iron. This is potentially damaging the endothelial wall and thereby contributing to cardiovascular disease and increased cardiovascular risk in conditions where increased iron levels circulate^9,10^. In patients with coronary artery disease, a lowering of iron, due to iron chelation, improved the endothelium, as measured by NO-dependent vasodilatation^11^. In addition, low doses of iron can quickly induce DNA damage responses in human endothelial cells^12^. In mouse models the NTBI fraction also played a crucial role in the exacerbation of atherosclerotic disease^13^.

There is limited research on the direct effects of iron on the vascular endothelium^10^. To investigate if and how different iron levels affect the endothelium, we investigated the role of increasing concentrations of iron on barrier integrity. In addition, we investigated the role of ROS in this process and tested for potential cooperativity with low grade inflammation induced by TNFα.

Our results show that FeCl_3_ has a dose-dependent negative effect on endothelial barrier integrity of Human Umbilical Vein Endothelial Cells (HUVEC), which can be prevented by the addition of ROS scavengers and inhibition of ROCK (Rho Kinase), a kinase that drives actomyosin-based contraction. In addition, we found that FeCl_3_ increased the protein levels of the small GTPase RhoB, a stress-responsive protein and inducer of EC contraction and vascular permeability. Finally, we found additive effects of FeCl_3_ and TNFα, suggestive for a pro-inflammatory role of circulating, free iron.

## Methods

### Antibodies and reagents

The following antibodies were used in this study: anti-ICAM-1 (Santa Cruz, sc-8439), anti-VE-Cadherin (XP Cell signaling D87FZ 2500 s), anti-RhoB (SC180 lot#10,416), GAPDH (Cell signaling 14c10, 2118 s), anti-caspase 9 (#9502, Cell Signaling Technology), anti-RhoA (#2117, Cell Signaling Technology), anti-claudin-5 (#34–1600, Invitrogen).

The following reagents were used: N-acetyl-L-cysteine (Sigma Aldrich), Ascorbid acid (Sigma life Science), Y27632 (Tocris bioscience), TNFα (Prepotech).

### Cell culture

HUVEC were purchased from Lonza and cultured on fibronectin (5 μg/ml) (Roche)-coated T75 plates at 37 °C in 5% CO2 atmosphere. ECM medium, supplemented with SingleQuots including 5% Fetal Bovine Serum (Lonza), was used for culturing HUVEC. Cells were passaged twice a week and used for experiments until passage four.

### Iron

Iron III chloride powder was purchased from Fluka. It was prediluted in H_2_O to a 0.1 M stock. Iron(III) citrate (F3388, Sigma) and Iron(II) sulfate heptahydrate (F8633, Sigma) were both dissolved in water. Prior to treatment of the HUVEC, the stock solution was diluted 1:10 in medium without additional serum. In the medium, the FeCl_3_ will dissolve into free iron and chloride. Physiological and supraphysiological concentrations of 50, 100, 300 and 500 µM of FeCl_3_ were used. As a reference: normal values in human blood are between 12 and 30 µM Fe^3+^^14^

### Endothelial barrier integrity measurement

Measurement of the integrity of the endothelial barrier was performed using Electric cell-substrate Impedance sensing (ECIS). For this assay, HUVEC were seeded in fibronectin-coated 96-well ECIS slides (Applied Biophysics). Slides were mounted into the ECIS instrument, and the resistance of the electrodes was monitored in real time at a frequency of 4000 Hz at 37 °C during next 100 h. At 48 h following seeding, cells formed a stable monolayer (resistance values were 1500–1700 Ohm), and resistance was measured in real time to compare the barrier integrity among different treatments. Addition of FeCl_3_ in the absence of cells did not induce any detectable effect on the ECIS electrodes (supplementary data; Fig. S1).

### Immunoblotting

Cell lysates for immunoblotting were collected in lysis buffer (10 mM Tris/Cl pH 7.5, 150 mM NaCl, 0.5 mM EDTA, 0.5% Nonidet™ P40 Substitute, 0.09% sodium azide). Proteins were separated using SDS-PAGE on 7.5% or 12.5% polyacrylamide gels and transferred to the nitrocellulose membrane. Membranes were blocked in 5% BSA in TBST-T for 1 h at 25ºC and probed with primary antibodies diluted in the blocking buffer overnight at 4 °C. Proteins were visualized using secondary anti–rabbit or anti–mouse antibodies coupled to HRP followed by enhanced chemiluminescence (Amersham/GE-healthcare) on AI-600 machine. Densitometric analysis of the band intensities was performed using ImageQuant.

### Immunofluorescence microscopy of cultured HUVEC

HUVEC were seeded on fibronectin-coated 2 cm^2^ coverslips (Thermo scientific, Menzel-gläser). Confluent cells were used for experiments, following a change of medium every other day. Cell fixation was performed with warm (37ºC) 4% paraformaldehyde (Sigma Aldrich) in phosphate buffered saline (PBS) (B. Braun) and incubated for 15 min at room temperature. PFA was washed away with PBS, cells were permeabilized with 0.2% triton X-100 in PBS for 3 min and blocked for 30 min with 1% HSA in PBS. Next, coverslips were stained with primary antibodies in 1%HSA/PBS overnight at 4ºC. After washing with PBS, the coverslips were incubated with the secondary antibody which was FITC-labeled (anti-rabbit or anti-mouse 1:100), F-actin-stain Alexa-670 phalloidin and DAPI (Thermo Fischer Scientific) for 1 h at room temperature. Next, the coverslips were mounted with MOwiol4-88/DABCO solution (Calbiochem, sigma Aldrich). Images were taken using a Nikon A1R laser-scanning confocal microscope.

### Measurement of intracellular ROS

After treatment with FeCl_3_ for 24 h as indicated, HUVEC were washed once with PBS supplemented with 1 mM CaCl_2_ and 0.5 mM MgCl_2_. Intracellular ROS were stained with 5 µM 2',7'-dichlorodihydrofluorescein diacetate (H2DCFDA; D399, Thermo Fisher Scientific) for 30 min at 37 °C. Hereafter, HUVEC were washed three times with PBS supplemented with 1 mM CaCl_2_ and 0.5 mM MgCl_2_ and fluorescence intensity was measured in a fluorescence plate reader (SpectraMax iD3, Molecular Devices). Fluorescence intensity was normalized to the average fluorescence intensity of the control after subtracting background fluorescence intensity.

### Overnight stimulation with TNFα

For overnight stimulation with TNFα, HUVEC were seeded on a fibronectin-coated 12-well plate. After 48 h, the monolayers were exposed to different concentrations of FeCl_3_ and TNFα simultaneously. For simulation of low-grade inflammation, TNFα concentrations of 0.3 nM, 1 nM and 3 nM were used, and FeCl_3_ concentrations of 50, 100, 300 µM were used. Effects on endothelial integrity due to the co-stimulation were recorded using ECIS.

### siRNA transfection

HUVECs were seeded on fibronectin-coated ECIS or culture plates. When cells reached 70 to 80% confluency, siRNA transfection using Dharmafect reagent 1 (#T-2001, Dharmacon) in OptiMEM (Gibco) was performed. For gene silencing, a final concentration of 25 nM of ON-TARGET plus Human RhoB siRNA SMART pool (siRhoB) was used. ONTARGET plus nontargeting control pool (N.T.) was used as negative control.

### Statistical analysis

Data are presented as mean ± SD. Student t-test was performed to compare 2 conditions. Comparison of more than one condition was tested by one-way ANOVA. P-values were considered statistically significant if *p* > 0.05. Statistical analyses were performed using GraphPad prism software.

## Results

### Iron induces a dose-dependent inflammatory response in HUVEC

**Figure 1:**
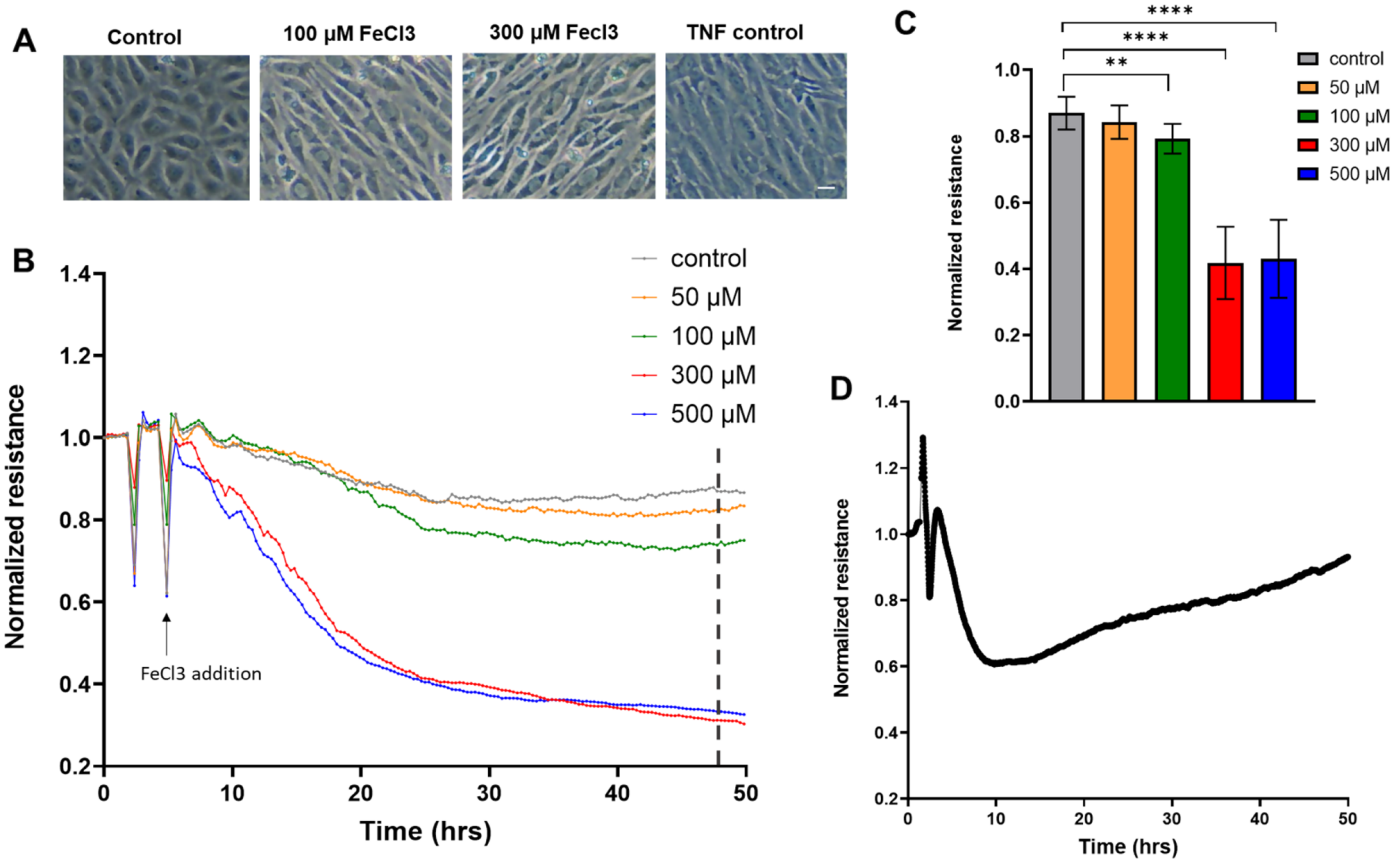
Iron-exposed HUVEC show morphological changes and a decrease in barrier integrity (**A**) Phase contrast images of HUVEC exposed to the indicated FeCl_3_ concentrations for 36 h. Note the elongation and alignment of the cells, as induced by both free iron as well as TNFα, included as a control, pro-inflammatory stimulus. Scale bar represents 50 µm. (**B**) HUVEC were cultured to confluency on fibronectin-coated 12-well or 96-well ECIS plates, followed by exposure to the indicated concentrations of FeCl_3_. At t = 6 h FeCl_3_ was added. ECIS analysis shows a dose-dependent loss of endothelial barrier function, induced by iron. (**C**) Quantification of ECIS data at 48 h (n = 3). (**D**) ECIS recordings of the response to TNFα (1 nM). Note that the TNFα response is transient, with a recovery after > 20 h. **P < 0.01, ****P < 0.0001. Data presented as mean ± SD. Comparison of 2 conditions was tested by student t-test.

To establish the effects of Fe^3+^ on endothelial integrity, we exposed HUVEC to physiological and supraphysiological concentrations of iron-III-chloride (FeCl_3_). Phase-contrast imaging showed that exposure to FeCl_3_ for 36 h induces morphological changes including marked elongation of the HUVEC. This resembles the response to TNFα, which is regarded as the golden standard for in vitro models of inflammation (Fig. 1A). Using ECIS as a quantitative means to assess the endothelial barrier, we found a dose-dependent drop in barrier function induced by FeCl_3_, with a ± 60% loss following 48 h exposure to the highest concentration of 500 µM (Fig. 1B,C,D shows TNF response as comparison). To test for the selective contribution of iron in this effect, we similarly treated HUVEC with FeCitrate or FeSO_4_ (Supplementary data, Fig. S2). This showed that while FeCitrate had a smaller and transient negative effect on the endothelial barrier as compared to FeCl_3_, FeSO_4_ (25–50 μM) induced a very pronounced loss of endothelial barrier function, with similar or even faster kinetics as compared to FeCl_3._

**Figure 2:**
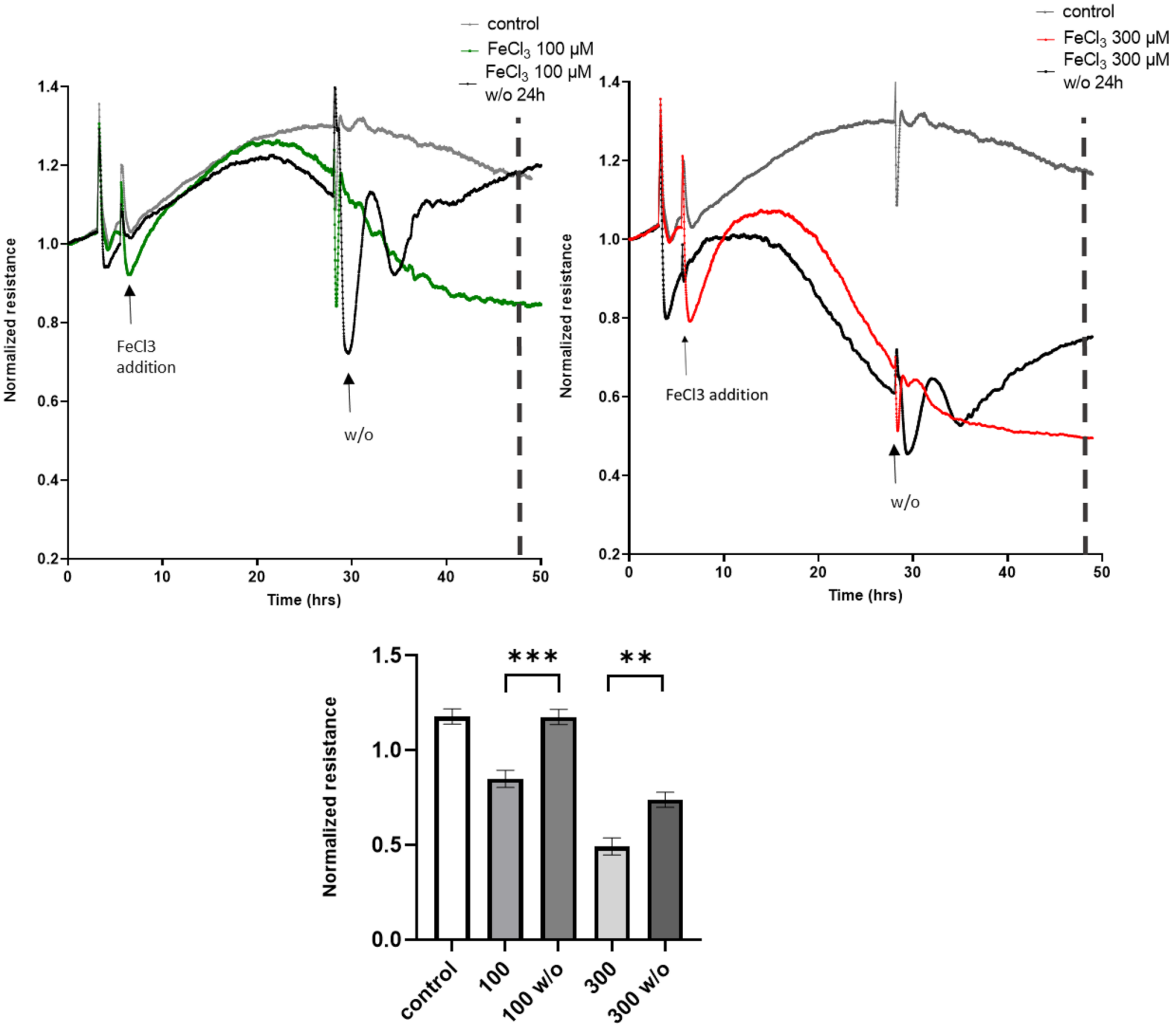
Wash-out (w/o) of FeCl_3_ after 24 h restores endothelial barrier integrity. HUVEC were cultured to confluency on fibronectin-coated 96-well ECIS plates followed by iron addition and wash-out of the indicated concentrations of FeCl_3_ (100 and 300 µM) which shows restoration of endothelial barrier integrity. At t = 5 h, iron was added to the HUVEC. Twenty-four hours after iron addition (t = 29) wash-out (w/o) of iron was done using complete ECM. Line graphs are representative for three individual experiments, Lower bar graph shows quantification of three experiments at t = 20 h after wash-out. **P < 0.01 ***P < 0.001. Data presented as mean ± SD. Comparison of 2 conditions was tested by student t-test.

When the FeCl_3_ was washed out after 24 h, the barrier recovered within 10–15 h (Fig. 2). This argues against the induction of cell death due to FeCl_3_ exposure. This was further confirmed with a western blot for caspase-9 levels in which no changes were found (Supplementary data, Fig. S3). Within this time-frame, barrier recovery was complete when cells were exposed 100 µM, and partial when cells were exposed to 300 µM FeCl_3_. This shows that the effect of FeCl_3_ is largely reversible and that barrier loss is not the result of EC apoptosis or cell death.

**Figure 3:**
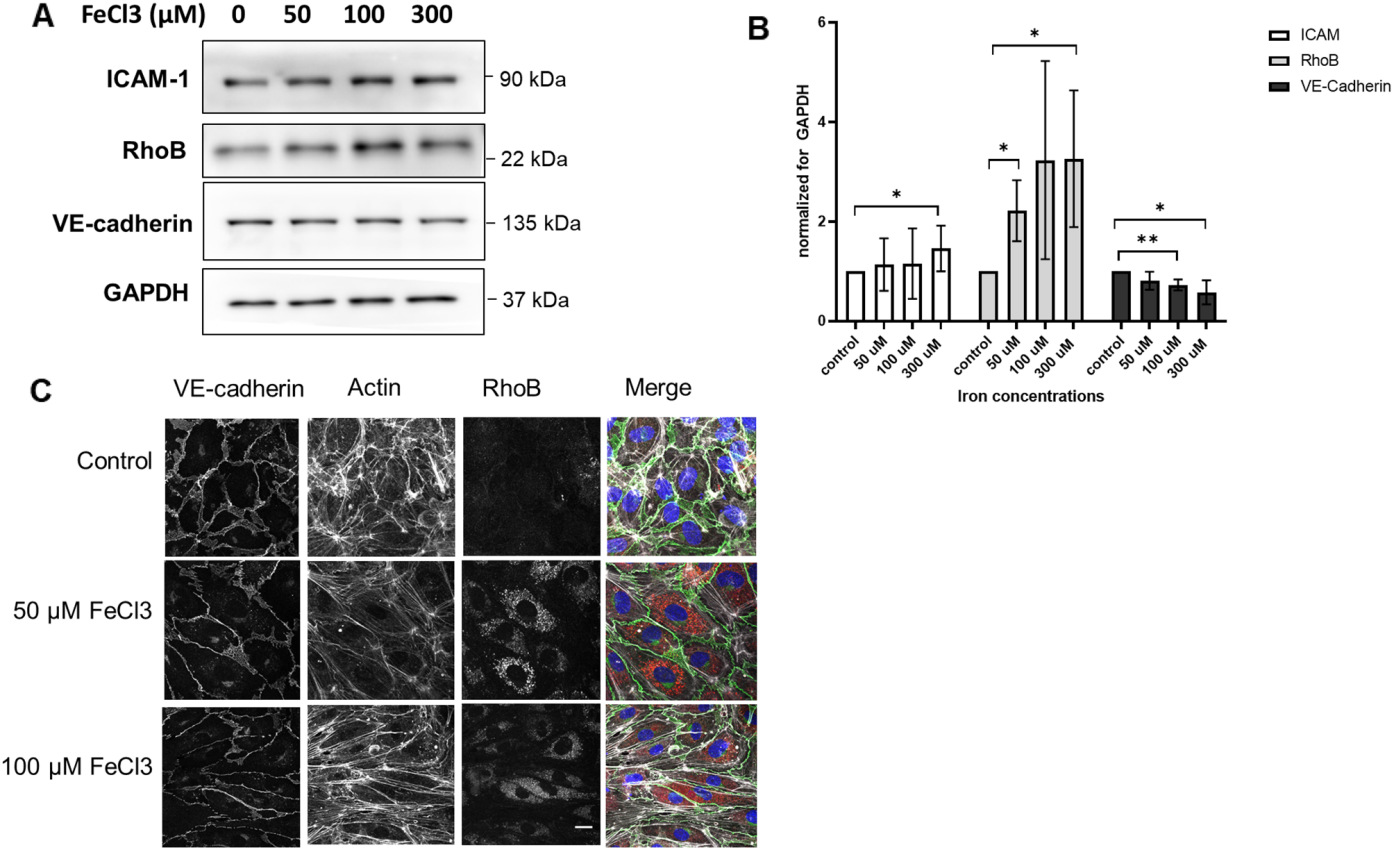
Changes in protein expression in iron-exposed HUVEC. (**A**) Western blot analysis of iron-exposed HUVEC for 48 h; (**B**) Quantification of western blots relative to GAPDH (n = 3), showing an increase in ICAM-1 (for 300 µM FeCl_3_) and RhoB (for 50 and 300 µM FeCl_3_) protein levels. And a small, but significant, decrease in VE-Cadherin protein levels (100 and 300 µM FeCl_3_). (**C**) immunofluorescence microscopy images of HUVEC exposed to the indicated iron concentrations for 36 h followed by staining for VE-cadherin (green), F-Actin (white) and RhoB (red); nuclei are in blue. Individual channels are shown in grayscale to improve visibility. Scale bar represents 25 µm. Images are representative for three individual experiments. *P < 0.05 **P < 0.01. Data presented as mean with SD. Comparison of 2 conditions was tested by student t-test.

With immunofluorescence microscopy (IM) we could detect a small decrease in the levels of VE-cadherin at cell–cell contacts and could confirm the cell elongation (Fig. 3), as also detected by phase contrast imaging (Fig. 1A). In addition, we tested for effects on the expression of the inflammation-associated and stress-responsive small GTPase RhoB, of which we know that it negatively controls endothelial integrity^15,16^. Following the exposure to FeCl_3_ (50 µM and higher), we could detect a clear increase in the protein levels of RhoB, which localizes to cytoplasmic vesicles, in particular in the perinuclear region^16^ (Fig. 3). Western blot confirmed the imaging data and showed a dose-dependent upregulation of RhoB, induced by FeCl_3._ RhoA levels did not show any changes (data not shown) following FeCl_3_ exposure. VE-Cadherin protein levels were downregulated to a limited extent (Fig. 3A,B), which is also in line with the microcopy results. Protein levels of claudin-5, a tight junction protein^17^, were also checked, but no changes in its levels were observed (Supplementary data, Fig. S3). We also analyzed changes in the level of the leukocyte adhesion receptor ICAM-1, which is also a marker for endothelial activation during inflammation^18^. However, we found that ICAM-1 upregulation was variable: some experiments showed a dose dependent increase, while others showed little to no change in ICAM-1 levels (Fig. 3A,B).

### ROS scavengers can partially prevent FeCl_3_-induced loss of barrier integrity

**Figure 4:**
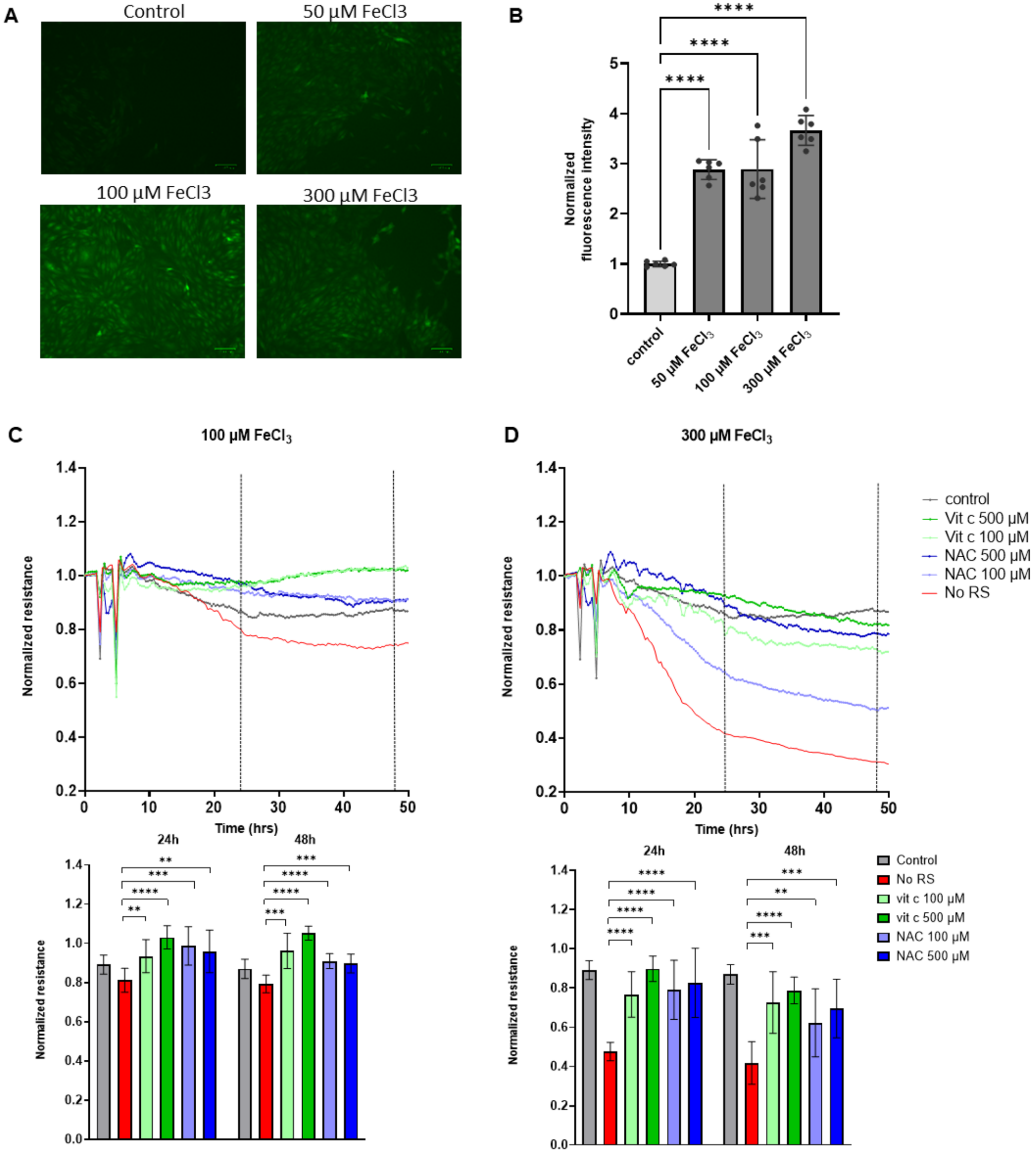
FeCl_3_ induced endothelial barrier changes in HUVEC can be prevented with ROS scavengers. (**A**) Microcopy pictures of 12 h iron treated HUVEC, loaded with the ROS sensitive dye DCFDA (**B**) Quantification of data shown in panel A (n = 3). (**C**, **D**) Upper panels: HUVEC were cultured to confluency on fibronectin-coated 96-well ECIS plates followed by exposure to 100 (**C**) or 300 (**D**) µM FeCl_3_ and 100 or 500 µM vitamin C/NAC. With the addition of these ROS scavengers (RS) an improvement of the endothelial barrier was seen. Lower panels: quantification of ECIS data (n = 3) at t = 24 and 48 h, indicated by the dashed lines. At 2 h, ROS scavengers were added, at 4 h FeCl_3_ was added. **P < 0.01, ***P < 0.001, **** < 0.0001. Data presented as mean ± SD. Comparison of 2 conditions was tested by student t-test.

**Figure 5:**
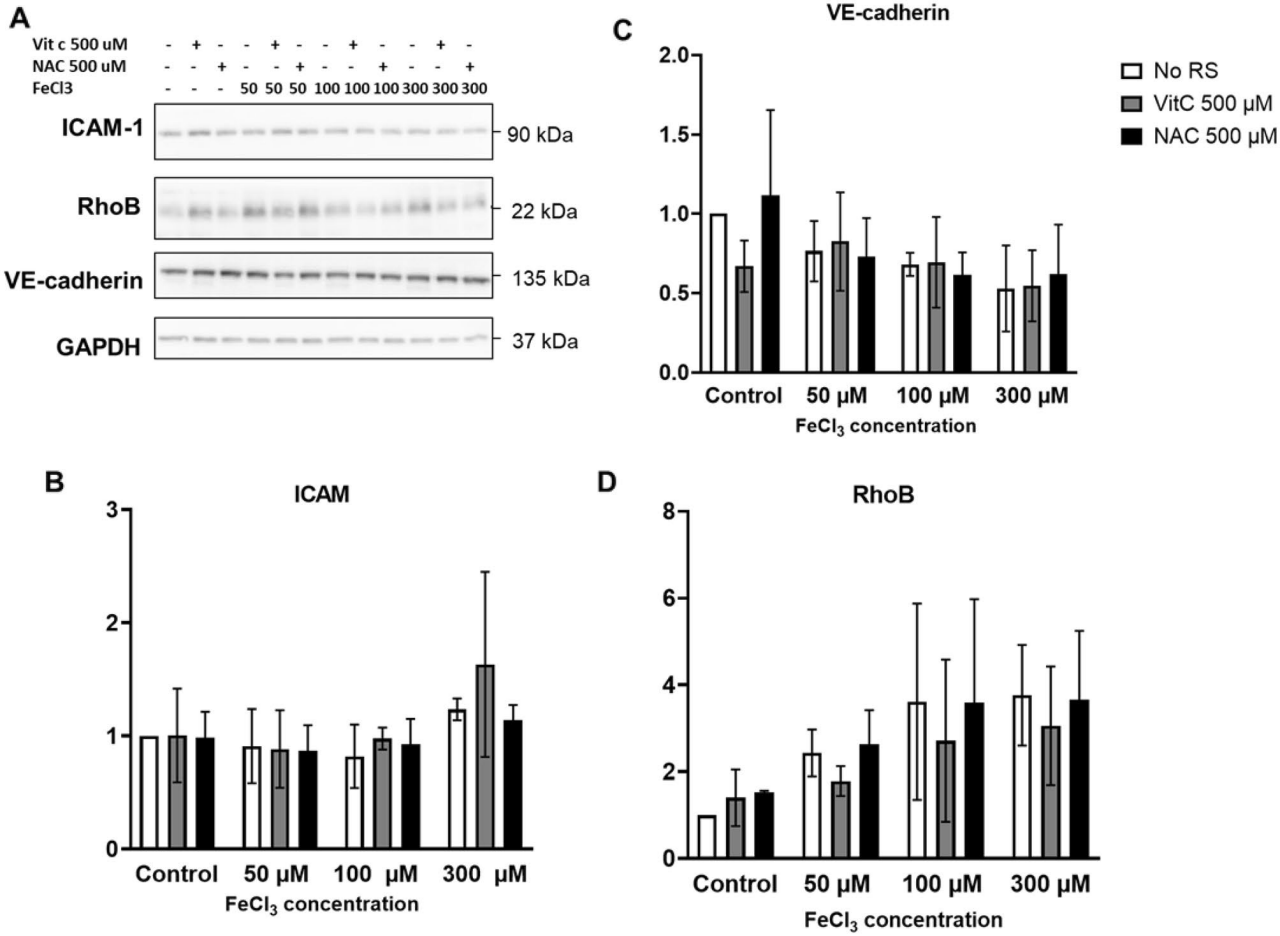
Protein expression following the addition of ROS scavengers (RS) to FeCl_3_-treated HUVEC for 48 h. (**A**) Western blot analysis for ICAM-1, RhoB, VE-Cadherin and GAPDH (loading control). (**B**, **C**, **D**) Quantification of western blots (n = 3) of ICAM-1, RhoB and VE-cadherin. Data presented as mean ± SD.

It is known from previously published findings that the damaging effect of iron on EC is accompanied by the production of Reactive Oxygen Species (ROS)^2,7^. We hypothesized that this was also related to the inflammation-mimicking response we observed in FeCl_3_-exposed HUVEC. In agreement with this notion, we found that addition of 50–300 μM FeCl3 induced the production of ROS, as detected using the ROS-sensitive dye DCFDA (Fig. 4A,B). To test whether these ROS were mediating the barrier-disrupting effects of FeCl_3_, we pre-treated HUVEC with two different ROS scavengers for 2 h: Ascorbic acid (vitamin C) and N-Acetyl-L-Cysteine (NAC), prior to exposure to increasing concentrations of FeCl_3_ and measuring barrier function by ECIS. Already in the absence of exogenously added FeCl_3_, a limited improvement in barrier integrity was induced by the different ROS scavengers, (Supplementary data, Fig. S4). For the FeCl_3_ concentration of 100 µM, the FeCl_3_-induced loss of endothelial integrity could almost completely be prevented by the ROS scavengers. For the concentration of 300 µM FeCl3, there was significant improvement of barrier function albeit that the ROS scavengers could not completely prevent the iron-induced loss of integrity (Fig. 4C,D). On WB, a small but consistent decrease in FeCl_3_-induced RhoB protein levels was seen following the addition of vitamin C but not NAC. Protein levels of ICAM-1 and VE-Cadherin did not show any differences following the addition of ROS scavengers (Fig. 5).

### ROCK inhibition induces additional improvement of barrier integrity

**Figure 6:**
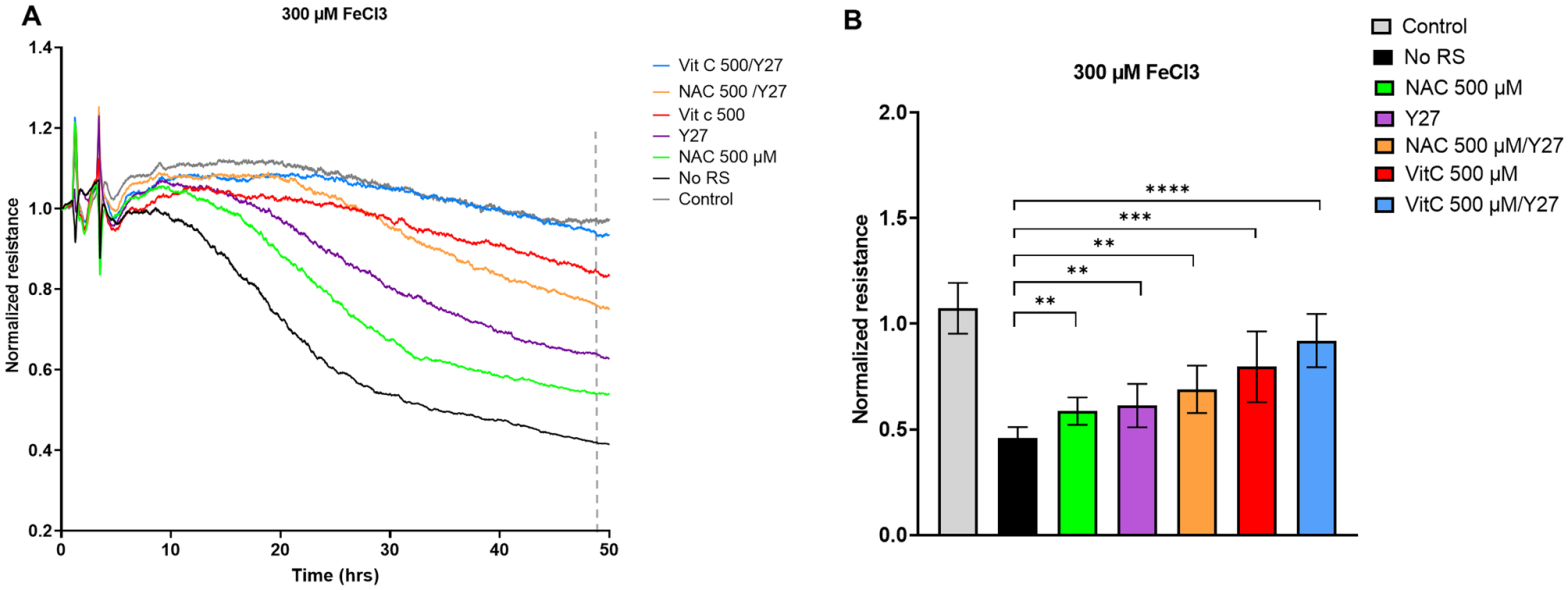
FeCl_3_ induced endothelial barrier changes in HUVEC can be further prevented with a combination of ROS scavengers (RS) and the ROCK inhibitor Y27632. (**A**) HUVEC were cultured to confluency on fibronectin-coated 96-well ECIS plates, followed by exposure to 300 µM FeCl_3_ and the RS vitamin C, at 500 µM (vit C), N-Acetyl-l-cysteine, at 500 µM (NAC), and/or the ROCK inhibitor Y27632 (Y27) at 10 µM. Graph is representative for 3 independent experiments (**B**) Quantification of ECIS (n = 3) at 48 h (dashed line). At 2 h, ROS scavengers/ROCK inhibitor were added, at 4 h FeCl_3_ was added. **P < 0.01, ***P < 0.001 **** < 0.0001. Data presented as mean ± SD. Comparison of 2 conditions was tested by student t-test.

Because of the upregulation of RhoB induced by FeCl_3_, and the fact that RhoB, similar to RhoA, is a potent inducer of ROCK-mediated F-actin stress fiber formation and contractility, we hypothesized that addition of a ROCK inhibitor would protect barrier integrity in the presence of FeCl_3_, likely via a different pathway than ROS scavenging. Figure 6 and Supplementary Fig. S5 show ECIS recordings of HUVEC that were pretreated for 2 h with ROS scavengers and the ROCK inhibitor Y27632 (10 µM) prior to FeCl_3_ addition. Similar to the ROS-scavengers, pre-incubation with Y27632 induced an improvement of barrier integrity, counteracting the barrier-disruptive effect of FeCl_3_. Combining ROS-scavengers and Y27632 showed a further, significant improvement of barrier integrity, with full recovery to control levels even in the presence of high concentrations FeCl_3_ (300 µM). Together, this data suggest that FeCl_3_ exposure induces a loss of endothelial integrity through the parallel induction of ROS production and of ROCK-mediated contractility**.** We were not able to unequivocally show that the RhoB GTPase was mediating this response (Supplementary data, Fig. S6).

### Low-dose TNFα synergizes with iron-mediated barrier loss

**Figure 7:**
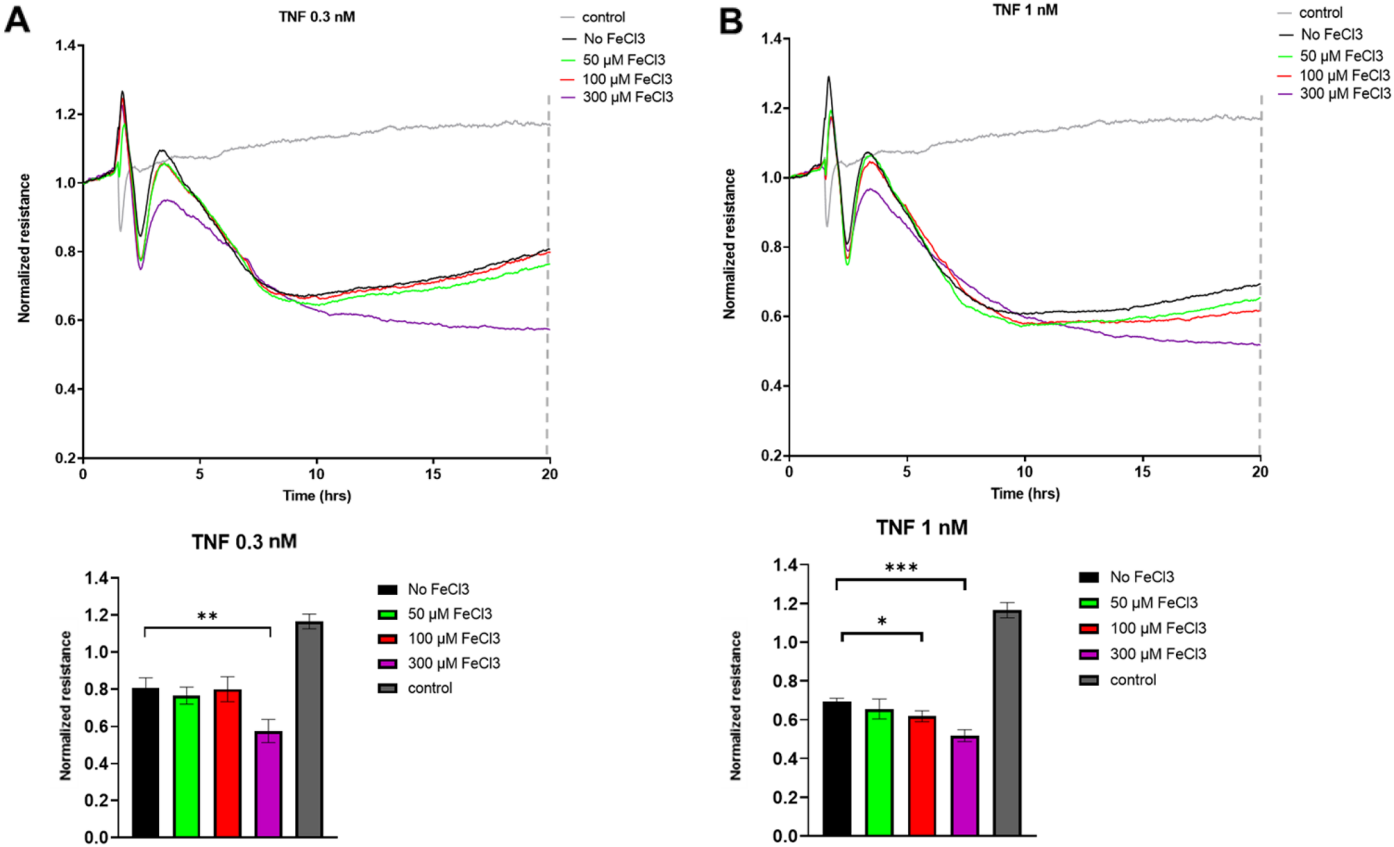
Co-stimulation of FeCl_3_ and low-dose TNFα shows a synergistic effect on endothelial barrier integrity. (**A**) HUVEC were cultured to confluency on fibronectin-coated 96-well ECIS plates, followed by exposure to the indicated concentrations of FeCl_3_ and TNFα. At t = 2 h, different concentrations FeCl_3_ and TNFα were added. Lower panel shows quantification of ECIS data at t = 20 h (**B**) HUVEC were cultured to confluency on fibronectin-coated 96-well ECIS plates, followed by exposure to the indicated concentrations of FeCl_3_ and TNFα. Lower panels show quantification of ECIS data at 20 h. At t = 2 h, different concentrations FeCl_3_ and TNFα were added. Graphs show data from a representative out of 3 independent experiments. Note that the same control is depicted in the graphs in (**A**, **B**). *P < 0.05, **P < 0.01, ***P < 0.001. Data presented as mean with SD. Comparison of 2 conditions was tested by student t-test.

The above findings suggest that FeCl_3_ induces, to some extent, a pro-inflammatory state in EC. To investigate this further, co-stimulation with FeCl_3_ and TNFα was performed. For this, suboptimal doses of TNFα were used (0.1, 0.3, 1, 3 nM) together with the FeCl_3_ concentrations used previously (50, 100, 300 µM). Endothelial barrier integrity decreased with all concentrations of TNFα. Moreover, an additional decrease with addition of 100 or 300 µM FeCl_3_ addition was seen. (Fig. 7 and Supplementary data, Fig. S7) On WB, ICAM-1 and RhoB protein levels showed a dose-dependent increase with both TNFα and FeCl_3_ (Supplementary data, Fig. S8). These data are suggestive for an additive effect of FeCl_3_ and TNFα on endothelial barrier disruption and on the protein levels of ICAM-1 and RhoB. These data suggest that low-to-moderate levels of circulating free iron can exaggerate a state of chronic, low-grade inflammation.

## Discussion

This study demonstrates a dose-dependent decrease in endothelial integrity, accompanied by a pro-inflammatory phenotype, induced by physiological concentrations of FeCl_3_ in concentrations mimicking male and female circulating iron levels^14^. This impaired integrity, accompanying endothelial activation, was gradually induced, reaching its maximum after 15–20 h and was reversible upon washout. We found that this response was accompanied by ROS production and that ROS scavengers (N-acetyl-L-cysteine and ascorbic acid) could, to a large extent, prevent FeCl_3_-induced loss of endothelial integrity. Similarly, inhibiting endothelial cell contraction using an inhibitor of ROCK, also partially prevented iron-induced loss of integrity. Most importantly, combining ROS scavenging and inhibition of ROCK completely prevented FeCl_3_-induced loss of integrity indicating that ROS and actomyosin-based contractility co-operate to mediated iron-induced loss of endothelial barrier function. It is important to note that while, among different experiments, the quantitative effects of the ROS scavengers and ROCK inhibitor varied, their qualitative effects as well as their combined inhibition of barrier loss was consistent throughout the entire study.

In addition to the effects on barrier function and in line with the accompanying pro-inflammatory phenotype, we discovered an FeCl_3_-induced increase in the protein levels of the Rho GTPase RhoB, but not its closely related homologue RhoA. RhoB is a TNFα-induced, stress responsive protein, which stimulates ROCK-mediated cell contraction and loss of endothelial barrier integrity^15,16^. With immunofluorescence microscopy, we found that the intracellular distribution of RhoB mimics the response to TNFα, which is characterized by a marked accumulation of RhoB in perinuclear vesicles^16^. Scavenging ROS with ascorbic acid prevented the iron-induced increase in RhoB protein. While we could not firmly establish that RhoB specifically mediates the observed loss of barrier function, its upregulation is indicative for the induction of cellular stress by iron.

TNFα-induced inflammation is also characterized by a strong increase in expression of the leukocyte adhesion molecule ICAM-1^19^. While exposure to FeCl_3_ by itself only slightly upregulated ICAM-1, we found that co-stimulation with increasing concentrations of FeCl_3_ and TNFα showed a synergistic effect on both ICAM-1 and RhoB expression. Similarly, an additional loss of endothelial integrity was found when monolayers were co-stimulated with FeCl_3_ and TNFα. These data show that FeCl_3_ by itself induces a limited pro-inflammatory state, which can be further increased by (suboptimal) doses of TNFα. In line with this, when HUVEC were exposed to serum from patients with iron overload (due to hemochromatosis or transfusion-dependent iron overload), this induced an increase in soluble ICAM-1^20,21^.

Our finding that FeCl_3_exposure by itself was not sufficient to significantly increase ICAM-1 suggests that iron, in contrast to TNFα, does not activate the NFκB pathway. Since the effects of FeCl_3_ could be significantly prevented by ROS scavenging, these data suggests that iron mainly acts via the induction of cellular stress pathways, rather than by inducing a change in transcriptional profile. Although TNFα is known to upregulate RhoB mRNA and protein, the effects of FeCl_3_ on RhoB levels may well be through a changes in RhoB protein turnover, as we and others have shown that RhoB has a short t_1/2_ and can be markedly regulated at the level of its ubiquitination and lysosomal degradation^16^. In this context it is interesting to note that various cellular systems and proteins are sensitive to iron, including ubiquitin ligases as well as lysosomes^22,23^.

Our findings are also in line with related data based on iron-II-citrate exposure of HUVEC, where an upregulation of inflammatory proteins was detected^12^. In HUVECs exposed to iron oxide nanoparticles a decrease in VE-cadherin protein levels was seen^24^. It is known that VE-cadherin is a key protein in endothelial cell–cell contact, and a major component of adherens junctions. The reduction in VE-cadherin protein could explain why we observed a dose-dependent, significant loss of barrier function. As proteolytic degradation of VE-cadherin does not appear to play a significant role, increased internalization of VE-Cadherin could explain the loss in integrity, the confirmation of which, however, would require additional in-depth analysis^25^.

Previous literature also showed an iron-induced increase in ROS in both EC culture and mouse model experiments^2,7^. The induction of oxidative stress following a 24 h exposure to iron was linked to the induction of apoptosis, and was accompanied by ROCK-mediated release of endothelial microparticles^26^. In our experiments, the effects of 24 h FeCl_3_ exposure on endothelial integrity were reversible, indicative for limited to no effects on cell death. Moreover, we could completely protect endothelial cells against the strong damaging effects of iron by simultaneously scavenging ROS and preventing ROCK-mediated contractility. Note that we formally cannot exclude a role for extracellular ROS in our experiments, although our data favor the conclusion that the observed effects are mediated by intracellular ROS.

In moderately iron-loaded mice, accelerated thrombus formation after arterial injury and increased vascular oxidative stress and reduced vasoreactivity was seen^27^. In animal models for thrombosis, high concentrations of iron (a solution of 30% FeCl_3_) are used to induce endothelial inflammation-driven thrombosis, which is also ROS-mediated^28^. This is in good agreement with our findings that show an inflammatory response to iron by the HUVEC. Thus, a small increase in free iron may cause increased, chronic endothelial activation, leading to inflammatory disease of the blood vessel wall, such as during atherosclerosis.

In vascular pathophysiology, iron has effects on many different cell types, not solely the endothelium. There is also a role for iron in macrophages where it leads to a more inflammatory phenotype, which can also contribute to the pathogenesis of atherosclerosis^13,29^. Thus, other factors should also be taken into consideration when assessing the effect of iron on vascular integrity and inflammation.

It is known that there are sex differences between males and females in the range of circulating iron with 10–25 µM in females and 14–35 µM in males^14^. In our study, the most clear effects of FeCl_3_ were seen using higher concentrations, as we observed a drop in barrier function and an increase in RhoB protein levels with 50–100 µM FeCl_3_. It is important to underscore that our in vitro conditions only partially mimic the human circulation, which makes it difficult to directly translate the effects on endothelial integrity, induced by these supraphysiological levels of iron. A related, important consideration, pertinent to the interpretation of our current findings, relates to the way in which the iron is presented to the EC. The HUVEC were exposed to FeCl_3_ (and FeSO_4,_ Fe_3_Citrate) in serum-containing medium, and therefore, a fraction of the added iron will become bound to the transferrin, which is in the serum. Given the amount of serum added (5% v/v) the amount of iron bound to transferrin will be limited^30^. Consequently, the remaining fraction of the added iron will be non-transferrin-bound (NTBI). Thus, these experiments model a situation of excess iron, where the available transferrin is saturated and the remaining iron is presented as partially bound to, e.g., albumin or citrate. Although we did not quantify which fraction of the iron that we used for treatment of the cells will be present as NTBI, our current findings and used concentrations of FeCl_3_ are in line with published studies in endothelial cells that addressed the effects of NTBI as a model for iron-induced cellular stress^31^. We therefore hypothesize that the effects we observed here are due to ‘free’, non-transferrin bound iron, which exerts pro-inflammatory effects on vascular endothelium.

We did not quantify in our experiments whether FeCl_3_ converted from its ferric (Fe^3+^) to its ferrous (Fe^2+^) form. Consequently, we cannot formally conclude that the effects of the added FeCl_3_ which we observed are due to ferric or ferrous iron. In endothelial cells, the divalent metal transporter (DMT1) transports ferrous iron into the cell^32^. It is possible that this also happens in our experiments and that ferric FeCl_3_ is converted to ferrous iron and transported by DMT1 into the EC. This could also explain the differences we observed in effective concentrations when investigating other iron forms (FeSO_4,_ Fe_3_Citrate). However, the in vivo conversion of ferric to ferrous iron and vice versa is a continuous process depending on the condition of the specific tissue. Therefore, quantification of ferrous and ferric iron is not an absolute requirement to allow drawing conclusions on the effects of iron on EC.

This is the first study that investigates barrier integrity in iron-exposed primary human EC. In vivo, EC are constantly exposed to low levels of iron. Together with the fact that iron plays a role in ROS formation in atherosclerotic plaques^7^, a small but consistent increase in iron could give a small but chronic loss of vascular barrier integrity which could eventually lead to significant EC activation and damage. This may be particularly relevant in situations of pre-existing cardiovascular inflammation, which we mimicked by combining iron with low-dose TNFα.

In conclusion, this study shows an iron-induced decrease in endothelial barrier function. This decrease in endothelial integrity could be prevented by the scavenging of ROS and the inhibition of ROCK-mediated contractility. On the protein level, an upregulation of stress-induced, pro-inflammatory proteins was detected, which could be partly prevented by ROS scavengers. These findings might have implications for our view on the role of iron as an endothelial activator. As we know that endothelial activation is a risk factor for cardiovascular disease in other diseases (e.g. systemic inflammatory diseases) this could also be translated to our views on sex and gender-related differences in cardiovascular risk, as iron and, consequently, endothelial activation differs between men and women.

### Supplementary Information

Supplementary Information.

## Data Availability

The data underlying this article are available in the article and in its online supplementary material.
