# Supplementary material for: The effect of circulating iron on barrier integrity of primary human endothelial cells
Source: Sci Rep. 2023 Oct 6;13:16857. doi: 10.1038/s41598-023-44122-6 (PMC10558552; doi:10.1038/s41598-023-44122-6)
Supplement: Supplementary file 1 — Supplementary Information. [file 41598_2023_44122_MOESM1_ESM.docx]

**
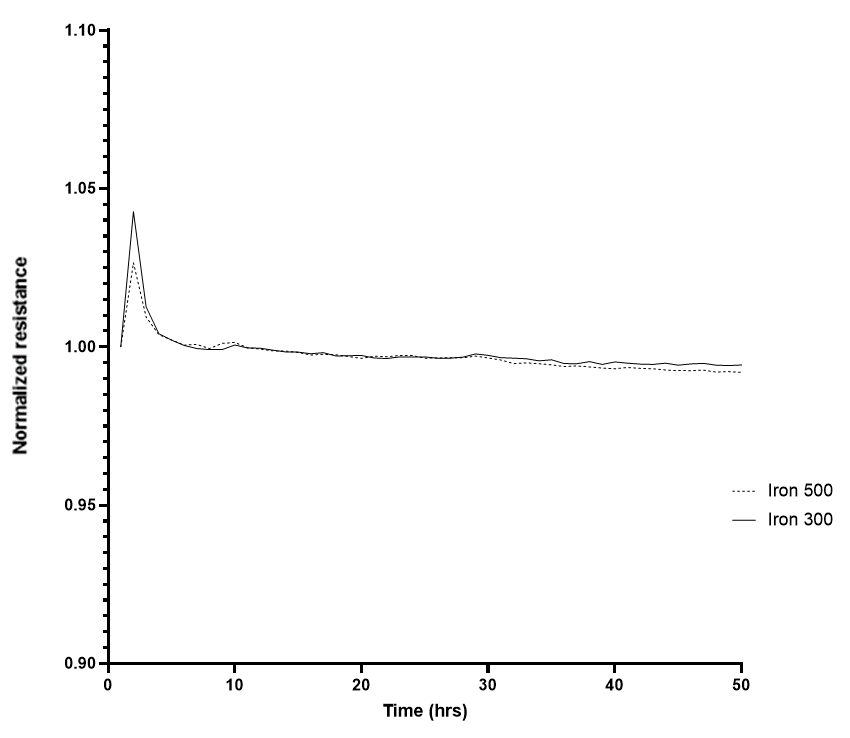
Madsen et al. Supplementary data, Figures S1-S9.**

**Supplementary Figure S1.** ECIS recording of an empty electrode (without HUVEC coverage) exposed to the indicated concentrations (in μM) of FeCl_3._ No effect of FeCl_3_ on the electrode and its basal impedance was detected.


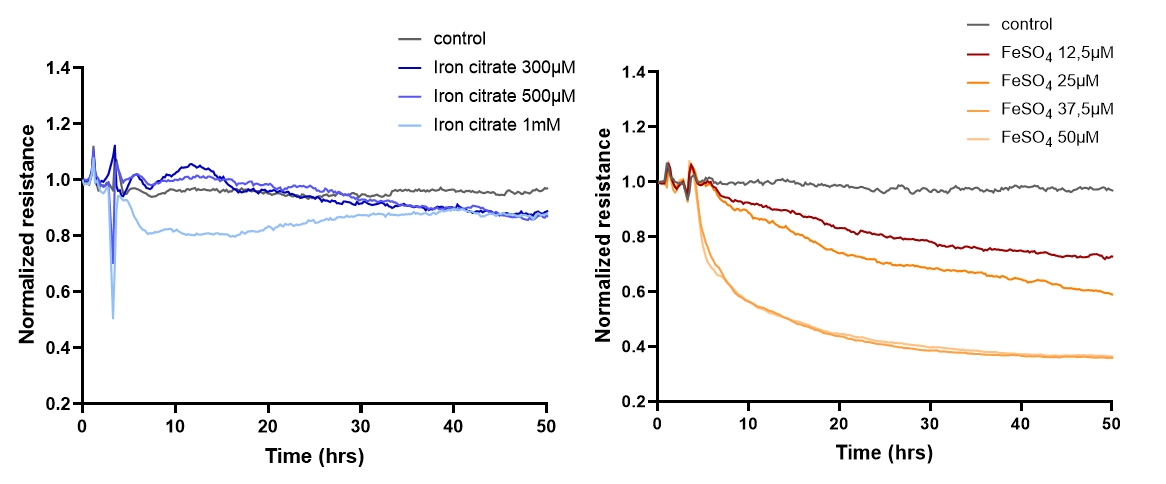


**Supplementary Figure S2** HUVEC were cultured to confluency on fibronectin-coated 96-well ECIS plates, followed by exposure to the indicated concentrations of iron citrate or FeSO_4_.


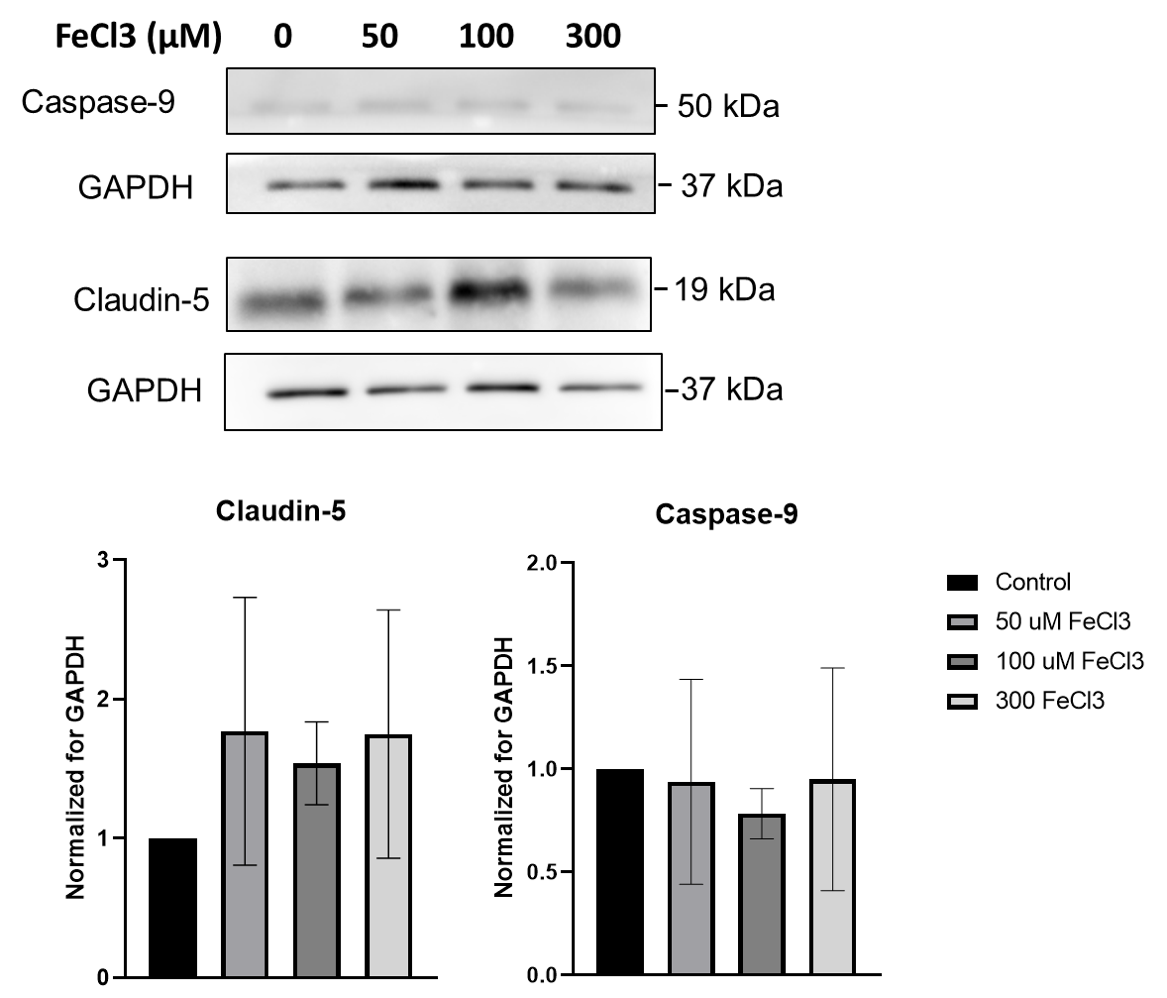


**Supplementary figure S3.** Protein levels of claudin-5 and caspase-9 do not show any significant changes following exposure of HUVEC to increasing concentrations of FeCl_3_. Upper panels, western blot analysis for caspase-9, claudin-5 and GAPDH (loading control). Lower panels: Quantification of western blots for Claudin-5 and Caspase-9 (n=3). HUVEC were cultured on fibronectin-coated 12-well plates. Data were normalized to GAPDH.

**
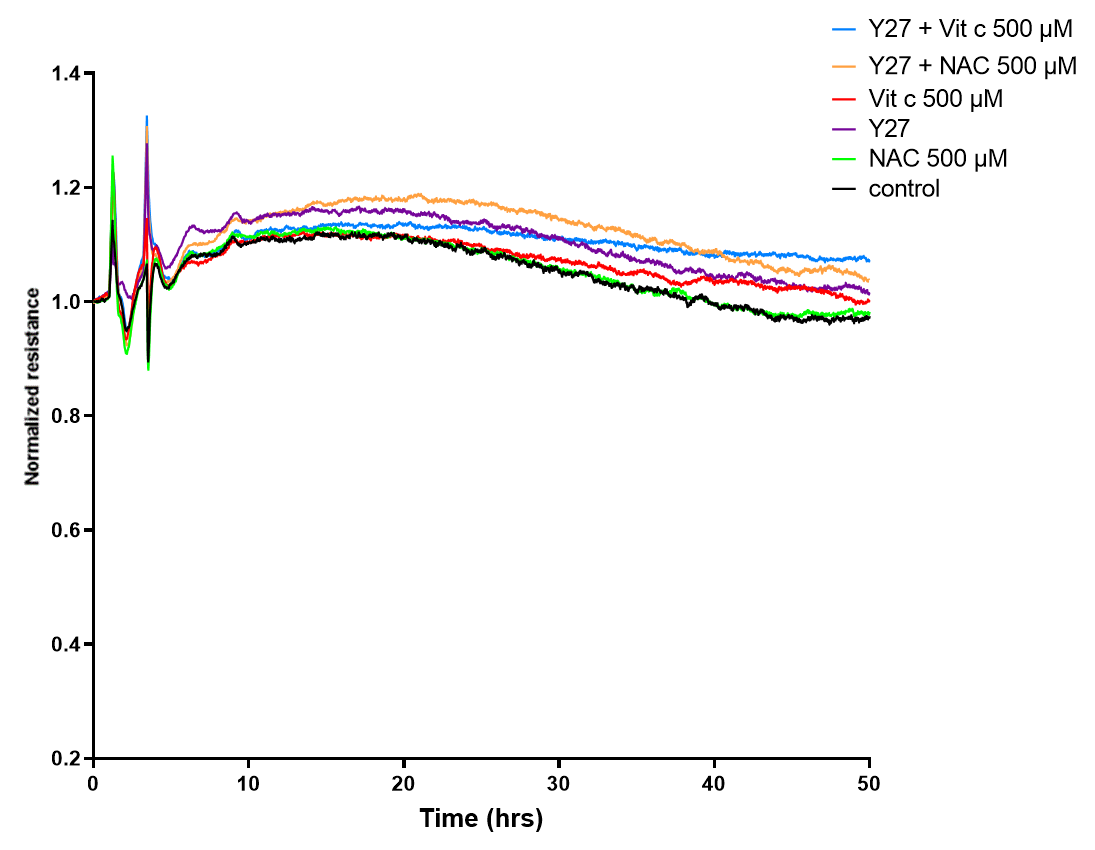
Supplementary Figure S4.** HUVEC were cultured to confluency on fibronectin-coated 96-well ECIS plates, followed by exposure to ROS scavengers vitamin C (Vit C, 500 µM), N-Acetyl-l-cysteine (NAC, 500 µM), and/or the ROCK inhibitor Y27632 (Y27, 10 µM). Graph is representative for 3 independent experiments.

**
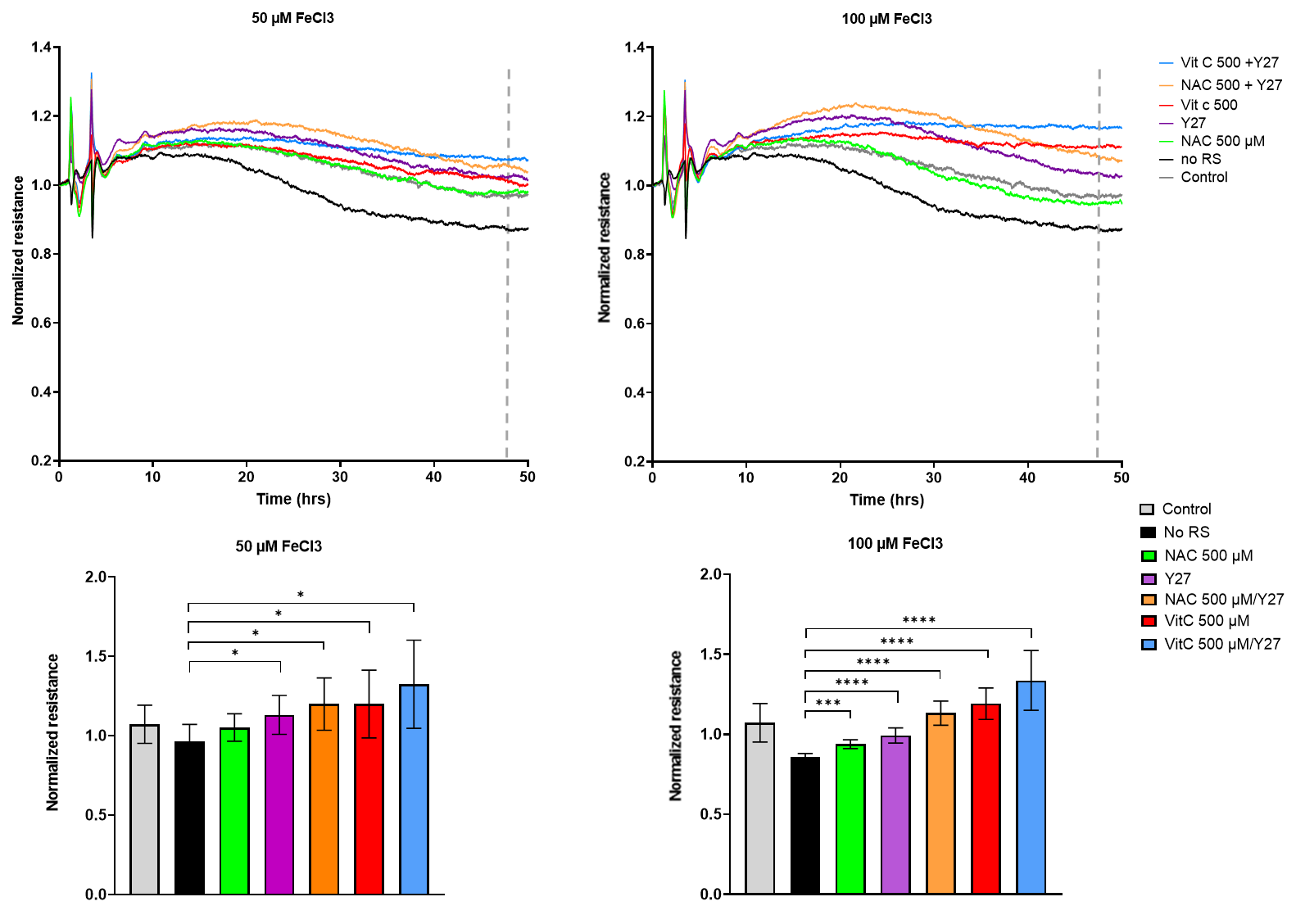
**

**Supplementary figure** **S5.** FeCl_3_-induced barrier changes in HUVEC can be further prevented with a combination of ROS scavengers and the ROCK inhibitor Y27632. HUVEC were cultured to confluency on fibronectin-coated 96-well ECIS plates, followed by exposure to 50 or 100 µM FeCl_3_ and ROS scavengers vitamin C 500 µM (Vit C), N-Acetyl-l-cysteine 500 µM (NAC), and/or the ROCK inhibitor Y27632 10 µM (Y27). Graph is representative for 3 individual experiments. Lower panel: Quantification of ECIS (n=3) at 48h. At 2h, ROS scavengers/ROCK inhibitor were added, at 4h FeCl_3_ was added. **P<0.01 ***P<0.001 ****<0.0001. Data are presented as mean + SD. Comparison of 2 conditions was tested by student t-test.

**
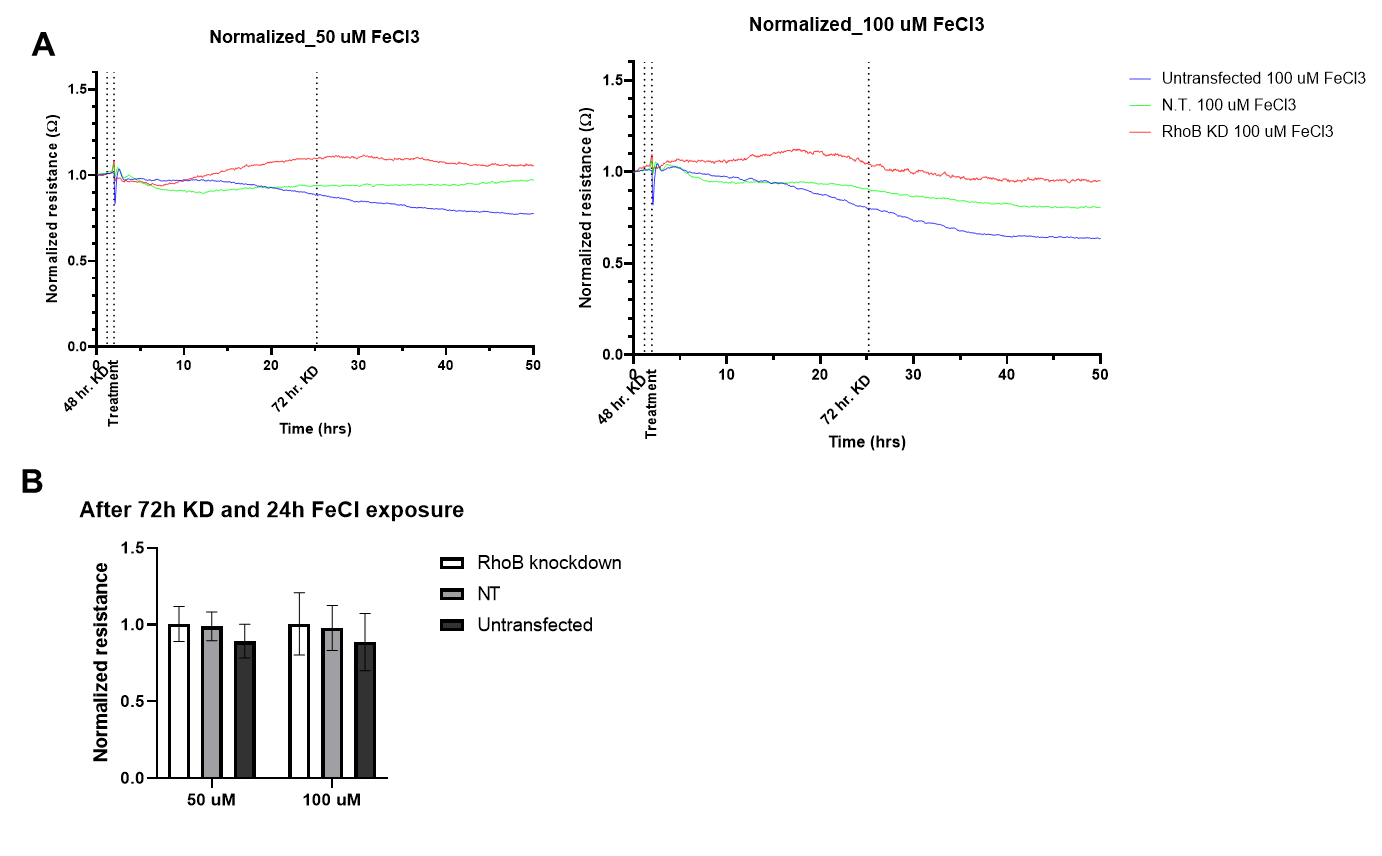
Supplementary Figure S6**. (A) ECIS experiments with knockdown of RhoB with siRNA combined with exposure to 50 and 100 µM FeCl_3_. Differences were seen between non-targeting (NT) and knockdown (KD) but this was not consistent between experiments as shown in the qualification of the optimal KD (after 72h) and maximal effect of iron exposure (after 24h).

**
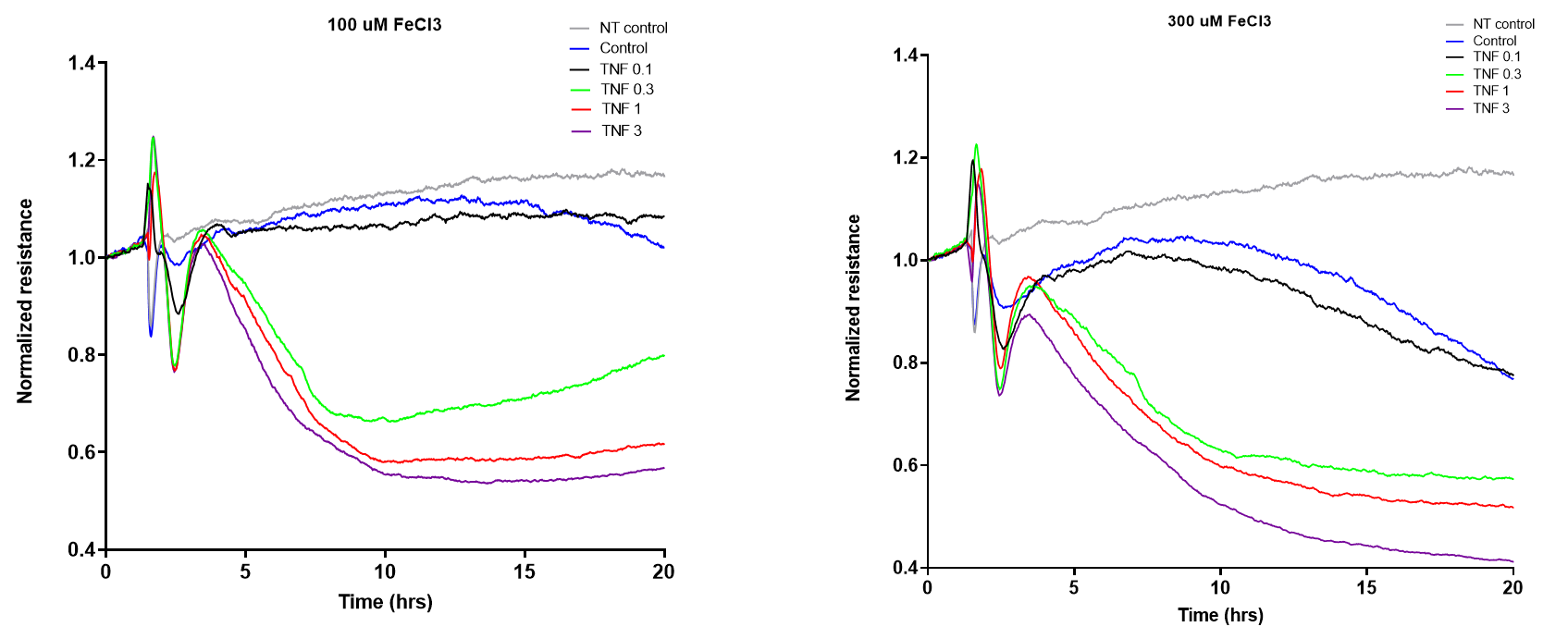
Supplementary figure S7. Co-stimulation of FeCl_3_ and low-dose TNFα shows an additive effect on endothelial barrier integrity.** HUVEC were cultured to confluency on fibronectin-coated 96-well ECIS plates, followed by exposure to the indicated concentrations of FeCl_3_ and TNFα. At t= 2h, the different concentrations of both FeCl_3_ and TNFα were added. Grey line shows untreated controls (NT), blue line shows only iron treated control.

**
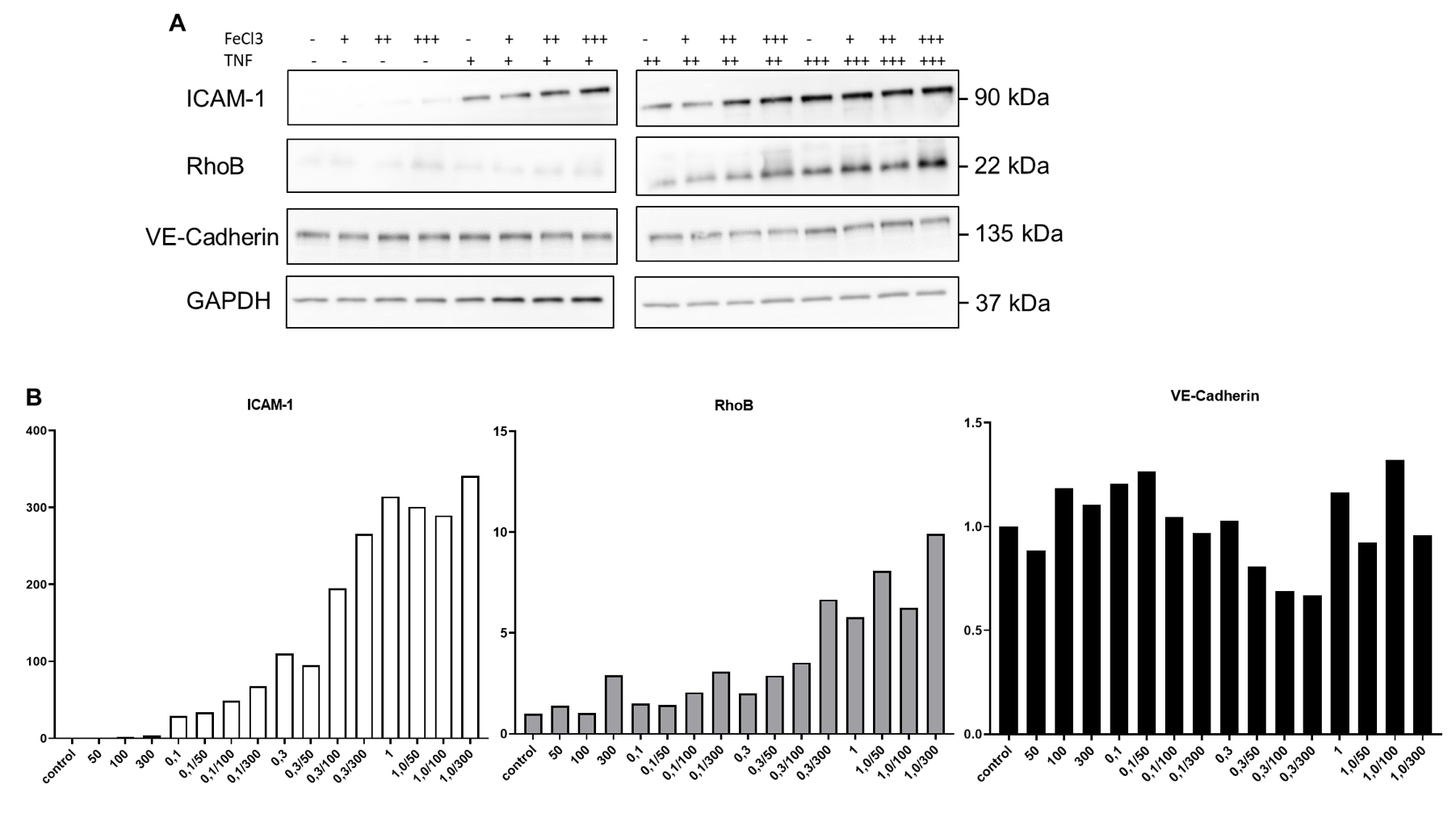
**

**Supplementary figure S8. Effect of iron on low-dose TNFα-exposed HUVEC on protein levels of ICAM-1 and RhoB for TNFα levels of 0.1, 0.3 nM and 1 nM. (**A) Western blot analysis for ICAM-1, RhoB, VE-Cadherin and GAPDH (loading control). (B) Quantification of western blots of ICAM-1 RhoB and VE-cadherin. HUVEC were cultured on fibronectin-coated 12-well plates. FeCl_3_ -= 0 µM +=50 µM ++=100 µM +++=300 µM TNFα -= 0 nM +=0.1 nM ++=0.3 nM +++= 1 nM. Data were normalized to control GAPDH due to technical problems with imaging of the higher concentrations of first blot.

**Supplementary data S9 Original blots**


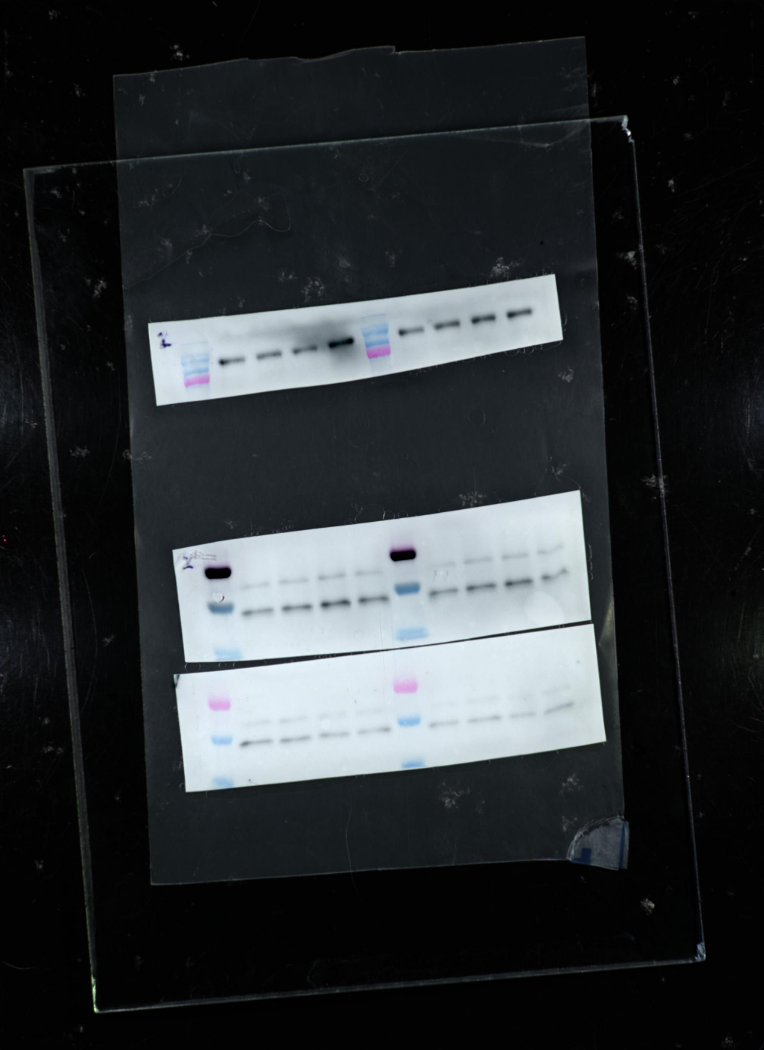
Original blots for Figure 3:

N=1

N=2

N=2

N=1

RhoB

VE-Cadherin

ICAM

GAPDH

GAPDH


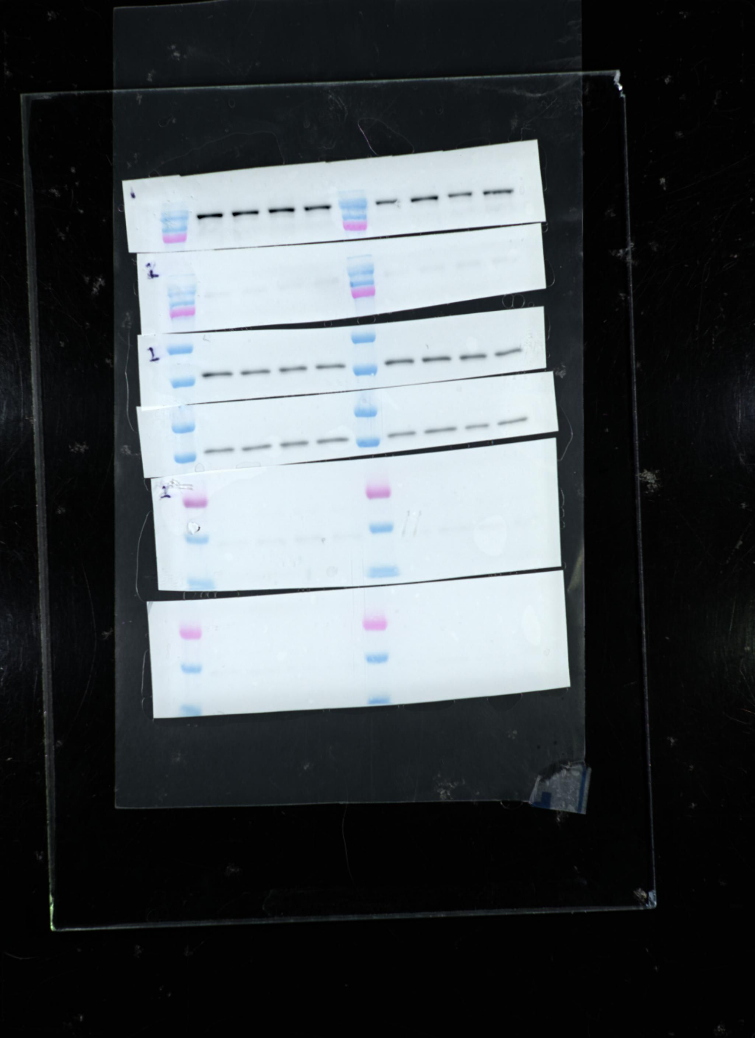


For the N=3 of this experiment the control lanes of the blots of figure 5 were used: 1^st^, 4^th^ 7^th^, 10^th^ lane.


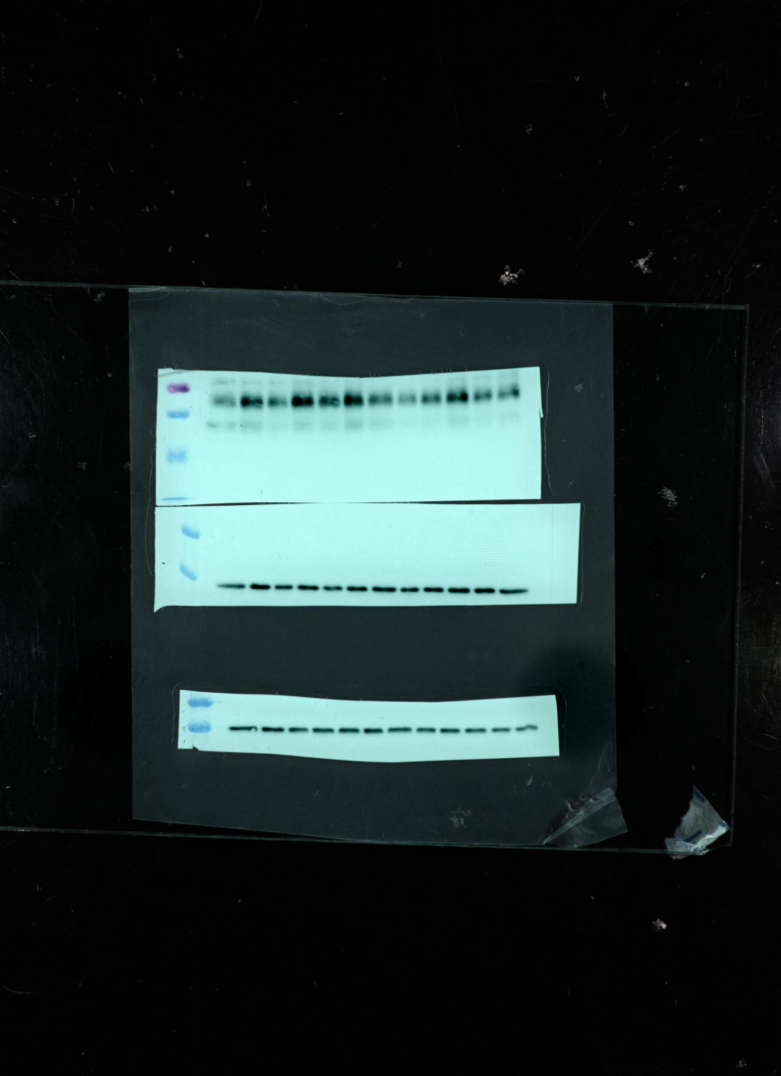

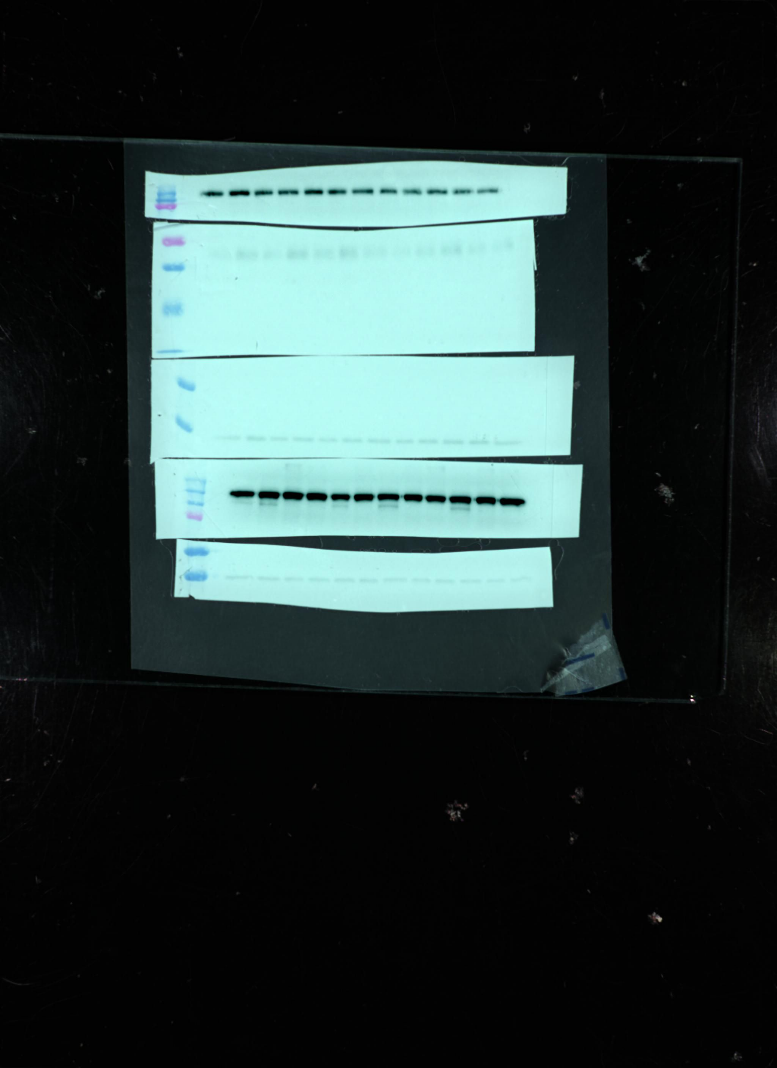
Original blots for Figure 5

N=1

N=1

RhoB

GAPDH

GAPDH

ICAM

VE-Cadherin


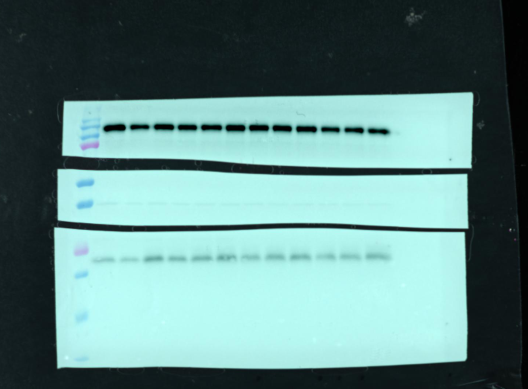

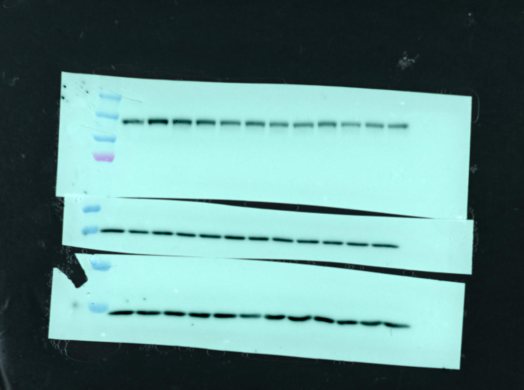


VE-Cadherin

N=2

N=2

RhoB

ICAM

GAPDH

GAPDH


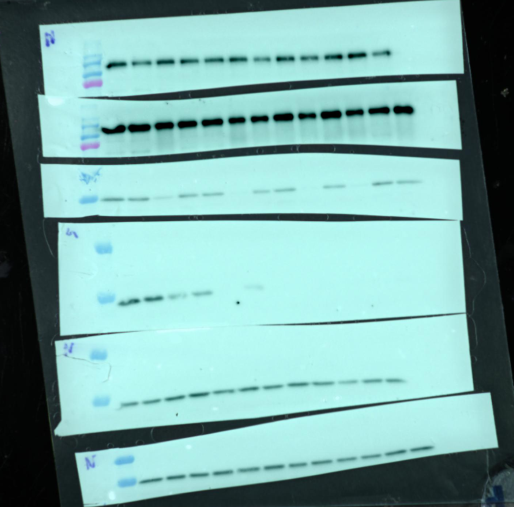


N=3

ICAM


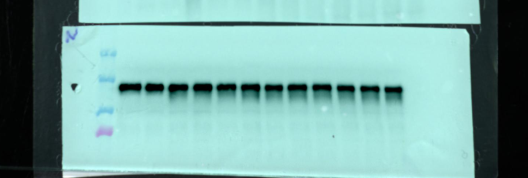

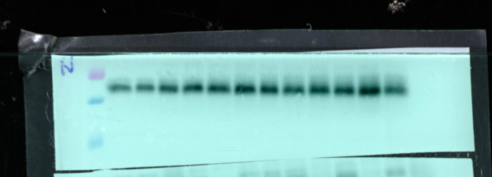

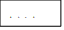


N=3

N=3

VE-Cadherin

RhoB

GAPDH

GAPDH

Original Western Blots Figure S3

Claudin-5


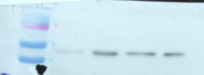

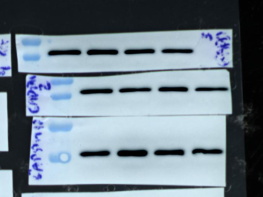

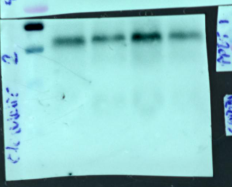

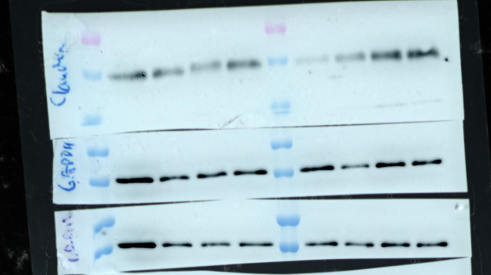

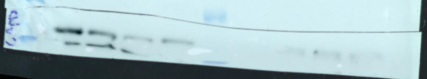


GAPDH

N=1

N=1

N=2

N=2

N=3

N=3

N=3

Caspase-9

GAPDH

Claudin-5

GAPDH

Caspase-9


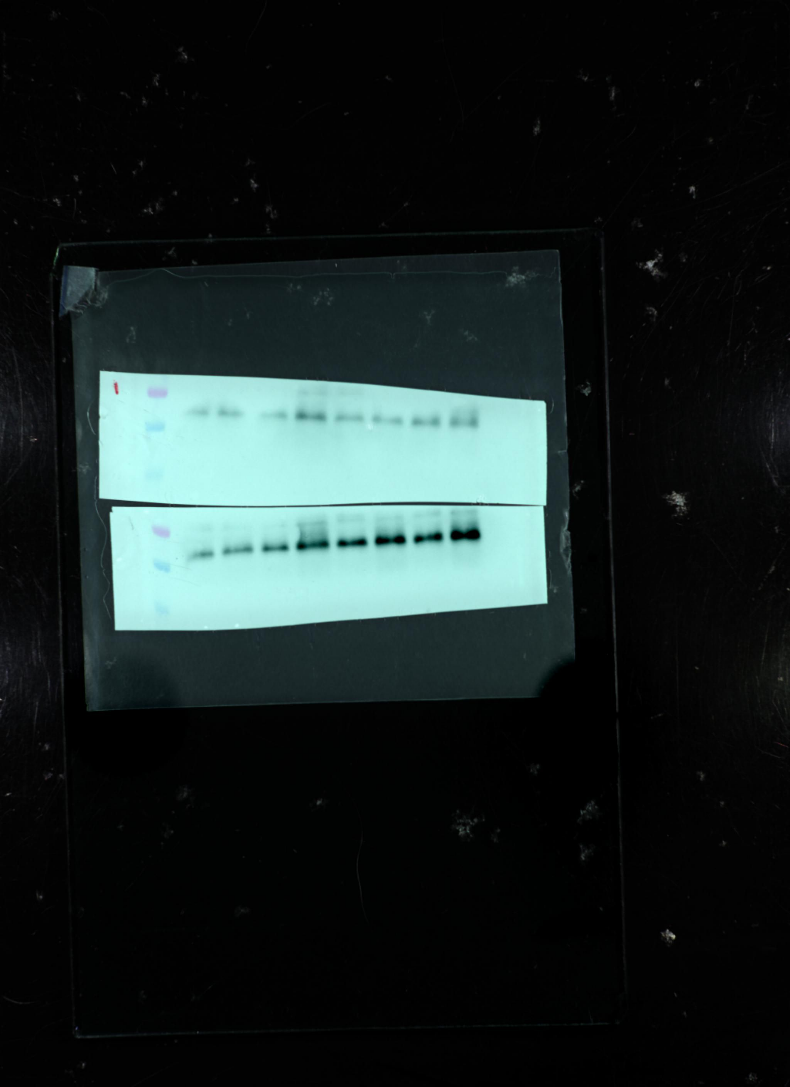

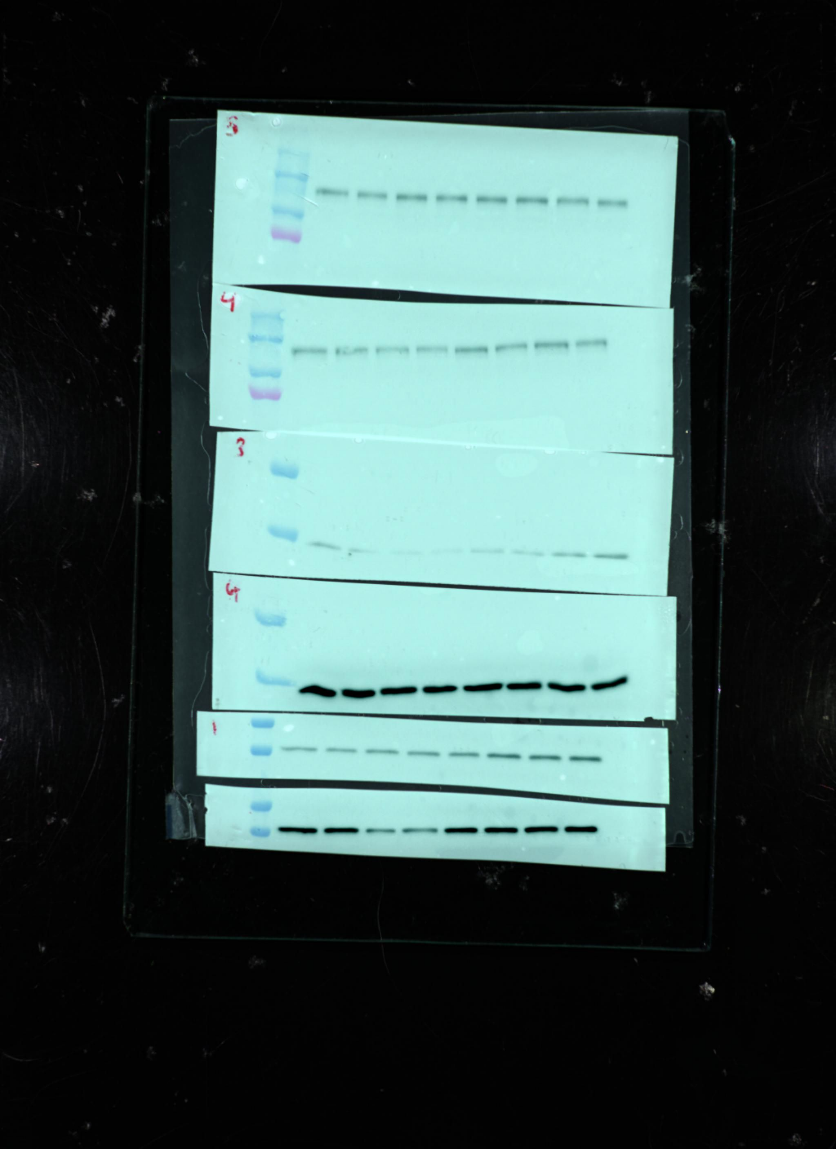

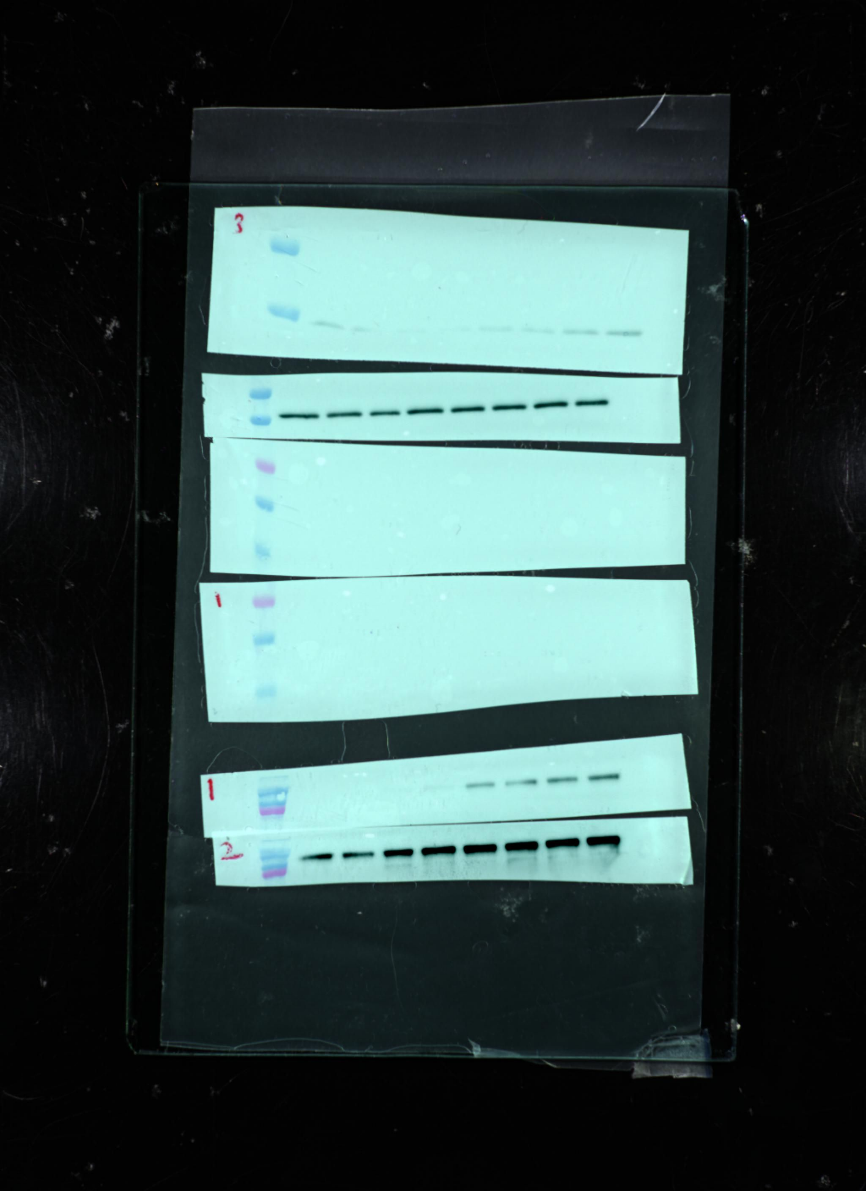
Original blots for Figure S8

N=1

N=1

GAPDH

GAPDH

GAPDH

GAPDH

GAPDH

ICAM

ICAM

VE-Cadherin

VE-Cadherin

RhoB

N=1

RhoB
